# Analog neural network–based wireless receiver

**DOI:** 10.1126/sciadv.aeg7153

**Published:** 2026-09-11

**Authors:** Chaoyi He, Yi Huang, Anil Korkmaz, Alireza Jaberi Rad, Qiangfei Xia, Linda Katehi

**Affiliations:** ^1^ Department of Electrical & Computer Engineering, Texas A&M University, College Station, TX 77843, USA.; ^2^ Department of Electrical & Computer Engineering, University of Massachusetts Amherst, Amherst, MA 01003, USA.

## Abstract

Traditional wireless receivers underperform in complex propagation environments and in the presence of nonlinear interference. Although data-driven artificial intelligence models offer a promising alternative, their implementation on conventional digital processing platforms is often limited by energy consumption and latency. Here, we present an analog wireless receiver that leverages the feature extraction and learning capabilities of neural networks for channel equalization, filtering, signal routing, and symbol detection. This modular architecture eliminates the traditional mixer for down-conversion and simplifies its implementation using analog in-memory computing hardware, allowing direct decoding of data from high-frequency analog signals. We demonstrate the adaptability, energy efficiency and processing speed of the proposed receiver through a proof-of-concept hardware implementation using memristor crossbar arrays on a system-on-a-chip platform. The fully hardware-implemented receiver achieves a bit error rate of 10^−6^ in detecting randomly generated symbols from distorted analog signals across multiple modulation schemes and carrier frequencies, highlighting its potential for future wireless communication systems for bandwidth-intensive and latency-sensitive applications.

## INTRODUCTION

The advent of the internet of things and the increasing demand for high-bandwidth wireless applications, such as autonomous vehicles, augmented reality, and swarm drones, have driven the exponential growth in the demand for adaptive and intelligent wireless communication systems. At the same time, the increasing complexity of wireless environments, characterized by diverse signal conditions and rapidly changing dynamics, is exposing fundamental limitations of traditional receivers in adaptability, energy efficiency, and processing speed. Addressing these challenges requires innovations in both signal-processing paradigms and hardware architectures that enable dynamic spectrum management, adaptive resource allocation, and efficient decision-making for next-generation wireless communication.

Artificial intelligence models have recently been introduced into wireless communication systems for adaptive signal processing. For instance, neural networks (NNs) have been applied to key receiver functions, such as channel estimation (*1*, *2*), interference mitigation (*3*), resource management (*4*), and modulation classification (*5*), due to their ability to learn and adapt to evolving signal conditions (*6*, *7*). These models have demonstrated strong performance in highly complex propagation environments, underscoring their potential for communication systems (*8*). However, most existing studies replace the entire receiver chain with a single deep NN (*9*–*11*). The resulting computational burden substantially increases power consumption and processing latency, posing substantial challenges for deploying deep NNs on traditional digital signal processors (DSPs), particularly in edge wireless systems.

From a hardware perspective, frequent data movement between memory and processing units in digital architectures further exacerbates energy consumption and latency, thereby limiting the real-time adaptability of NN-based receivers (*12*). Analog in-memory computing (AIMC) offers a transformative solution by fundamentally redefining the signal-processing paradigm (*13*, *14*). By directly processing analog signals and executing computations in memory, AIMC eliminates the von Neumann bottleneck associated with data transfer between separate memory and processing units (*15*–*18*). This approach has enabled early demonstrations of wireless signal processing, including spectrum analysis via discrete cosine transformation (*19*, *20*) and discrete Fourier transform (*21*, *22*), as well as in-phase and quadrature (I/Q) modulation and demodulation, experimentally implemented on memristor crossbar arrays (*23*, *24*) and the system-on-a-chip (SoC) platform (*21*). While existing wireless signal processing based on AIMC hardware highlights the potential for improved energy efficiency and reduced latency, these approaches remain constrained to use the traditional model-based (linear-algebraic) methods rather than data-driven NNs (*21*, *23*–*25*). Moreover, many prior studies focus primarily on experimental demonstrations of functional blocks of a wireless receiver and rely on simulations to evaluate overall system performance, lacking both system-level designs using AIMC and experimental validation of a wireless receiver (*26*–*29*).

Here, we present a wireless receiver architecture for low-power and low-latency edge processing. By embedding multiple lightweight NN models performing channel equalization, filtering, signal routing, and symbol detection, and exploiting AIMC hardware based on memristor crossbar arrays for energy-efficient implementation, the proposed design eliminates the need for a traditional mixer for frequency down-conversion and directly detects digital data symbols from high-frequency analog signals. Implemented on a multicore memristive SoC, the proposed receiver experimentally achieves a bit error rate (BER) of 10^−6^ at a 20-dB signal-to-noise ratio (SNR) across various modulation schemes and carrier frequencies, highlighting its adaptability and energy efficiency.

## RESULTS

### Analog NN-based wireless receiver

**Fig. 1.**
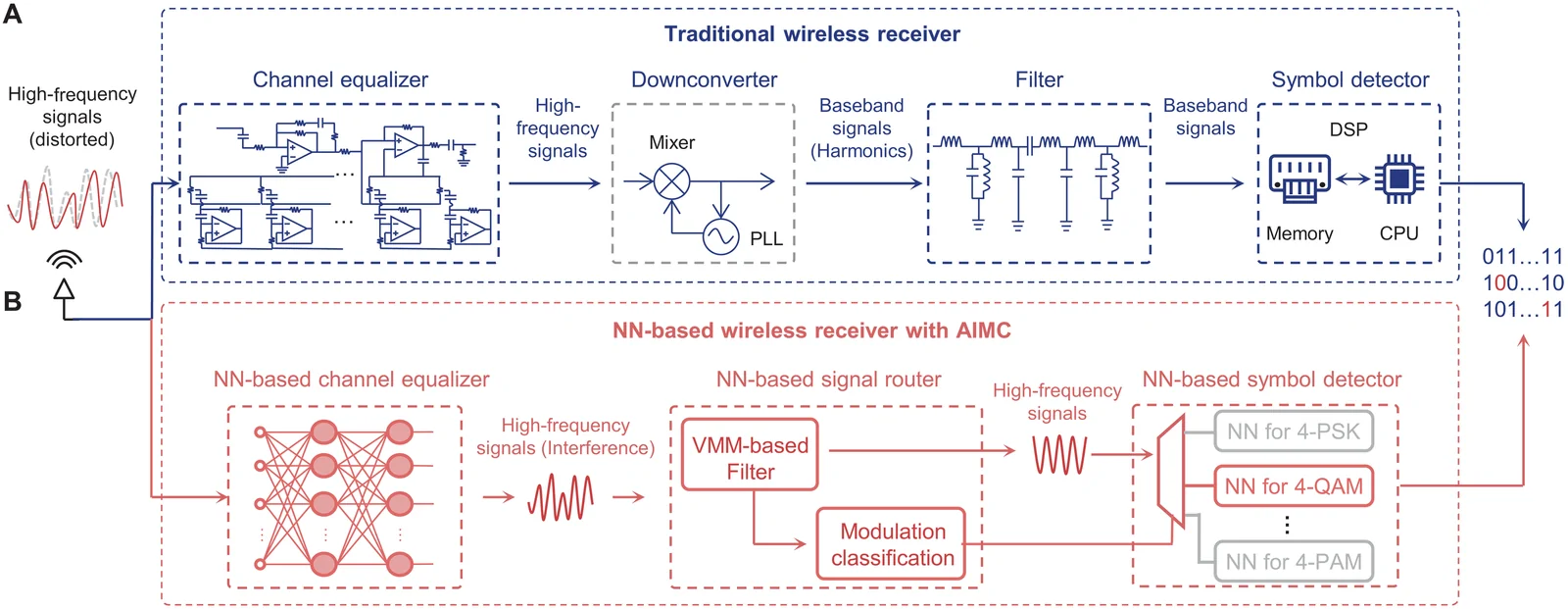
Traditional and analog NN-based wireless receiver. (**A**) Workflow and hardware implementation of a traditional wireless receiver. The received RF signal is first equalized with channel equalizer to mitigate channel-induced amplitude and phase distortions. It is then down-converted to baseband or an intermediate frequency using a mixer and a local oscillator synthesized by a phase-locked loop (PLL), and filtered by an analog baseband filter to suppress out-of-band noise and adjacent-channel interference. The resulting baseband signals are then digitalized by ADCs and processed in digital signal processors (DSP) to recover the transmitted symbols. (**B**) Proposed NN-based wireless receiver with AIMC hardware. The system comprises multiple NN-based modules, including: (i) an NN-based channel equalizer that learns and compensates for channel-induced amplitude, phase, and multipath distortions by extracting relevant channel features and performing adaptive equalization; (ii) a vector-matrix multiplication (VMM)–based analog filter that implements frequency-selective filtering through VMM in memristor crossbar arrays, suppressing out-of-band interference and adjacent-channel components; (iii) a modulation classifier (signal router) that identifies the modulation format and routes the filtered signal to the appropriate detection pathway; and (iv) a set of NN-based symbol detectors that directly maps high-frequency input waveforms to transmitted symbols, eliminating conventional demodulation, carrier recovery, and discrete-time DSP blocks. All modules are realized using memristor crossbar arrays that execute VMM operations in a single step. This approach improves energy efficiency, lowering latency, and enhancing adaptability for next-generation wireless systems.

In wireless data transmission, digital information is encoded and modulated onto a high-frequency analog carrier signal, which is then transmitted over wireless channels that inevitably introduce distortion. As shown in Fig. 1A, traditional receivers compensate for channel distortions in the received high-frequency analog signal using a linear channel equalizer, followed by a mixer for down-conversion to baseband. High-frequency harmonics introduced during this process are subsequently suppressed by analog filtering. The filtered analog signals are then converted to digital data by analog-to-digital converters (ADCs), after which symbol detection and information recovery are typically performed using DSPs. Such a signal-processing flow has limited applicability in dynamic environments, where rapid adaptation to changing channel conditions is required (*26*, *27*). Receivers today that use NNs for digital signal processing are typically trained to jointly perform multiple functions, such as synchronization, channel estimation, and symbol detection. However, although they exhibit strong adaptability, they incur substantially higher computational complexity than traditional receivers based on linear algebra and trellis-based algorithms. As a result, such approaches face fundamental challenges in energy efficiency and processing latency in edge wireless receivers, where computational resources are severely constrained.

To improve adaptability while maintaining low energy consumption and low latency, we propose an analog wireless receiver that integrates multiple NN-based signal processing modules to execute channel equalization, analog filtering, signal routing, and symbol detection. As shown in Fig. 1B, the received wireless signal is first processed by an NN-based channel equalizer to compensate for nonlinear distortions and mitigate the effects of additive white Gaussian noise (AWGN). After equalization, the signal passes through a finite impulse response (FIR) filter to isolate the desired frequency band and suppress out-of-band interference and noise. The filtered signal is then routed to an appropriate symbol detection NN by an intelligent router, which uses a modulation classification NN to identify the modulation scheme of the received signals. Unlike traditional receivers that require down-conversion mixers and complex compensation blocks to eliminate mixer-induced distortions when processing high-frequency signals (*28*, *29*), the proposed symbol detector operates directly on the high-frequency analog signals. This approach enables efficient symbol decoding while substantially reducing processing overhead. The modular NN architecture facilitates efficient hardware implementation of individual functional blocks using AIMC hardware based on memristor crossbar arrays, exploiting memristor reconfigurability and the inherent parallelism of analog vector-matrix multiplication (VMM; see Materials and Methods). To evaluate the system-level energy efficiency and latency of the proposed architecture, we distribute key components of the analog NN-based wireless receiver across multiple memristor crossbar arrays within the SoC platform (*21*).

### Feature extraction channel equalizer

**Fig. 2.**
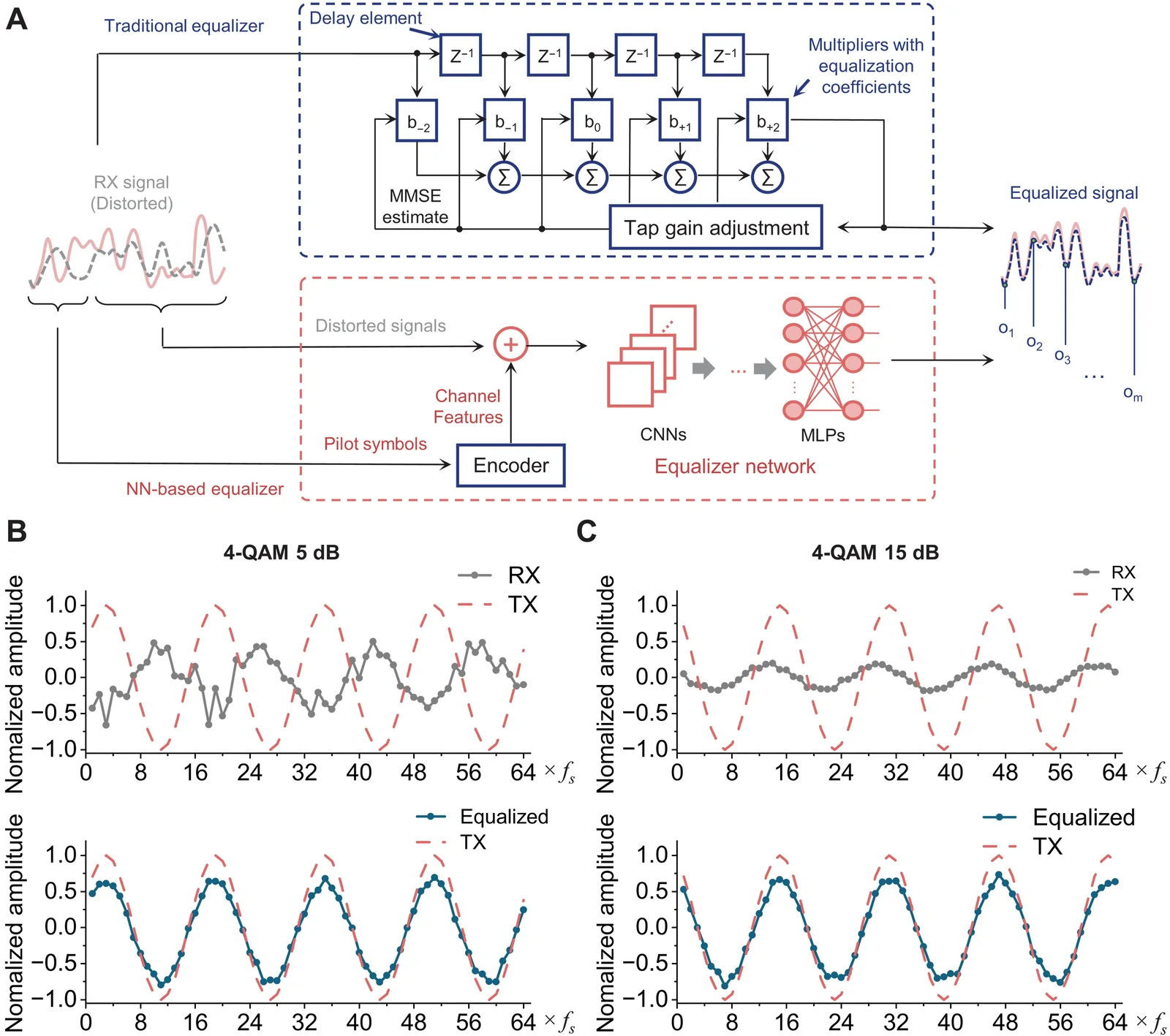
Feature extraction channel equalizer. (**A**) Comparison between traditional and NN-based channel equalizers. Conventional channel equalizers are typically derived from parametric signal models and linear estimation theory, which, while optimal under Gaussian noise and linear time-invariant channel assumptions, underperform in the presence of nonlinear distortions, and rapidly time-varying multipath channels. In contrast, NN-based equalizers adopt a data-driven framework. An encoder extracts channel-relevant features from pilot symbols, capturing both linear and nonlinear channel characteristics. These features are processed by a hybrid architecture combining convolutional neural networks (CNNs), which efficiently model local temporal correlations and multipath structure, and MLPs, which provide nonlinear mapping and global feature integration. (**B** and **C**) Comparison of distorted and equalized signals relative to the transmitter reference. After equalization, the recovered signals are compared with the original transmitter (TX) reference signals. The TX signals are modulated using four-level quadrature amplitude modulation (4-QAM) at SNRs of 5 and 15 dB, respectively.

As shown in the top flow of Fig. 2A, traditional channel equalizers, such as those based on minimum mean square error (MMSE), adjust the equalization coefficients to minimize the error between the received and transmitted signals (*30*). These channel equalizers rely on linear filters with delay elements and multipliers. While effective in simple environments, MMSE channel equalizers struggle in complicated channels due to limited adaptability and an inability to handle nonlinear distortions and complex noise profiles effectively. To address these challenges, we propose an NN-based channel equalizer that integrates a convolutional NN (CNN) and a multilayer perceptron (MLP) for adaptive equalization in dynamic wireless environments, as shown in the bottom flow of Fig. 2A. The NN-based channel equalizer utilizes pilot symbols, known reference signals embedded within the transmitted data, to extract detailed channel features. These features capture the dynamic characteristics of the communication channel, including nonlinear distortions and varying noise conditions. The extracted feature vector is concatenated with the raw received signal and processed by the NN-based channel equalizer, effectively compensating for both linear and nonlinear distortions.

Figure 2 (B and C) compares distorted (see Materials and Methods) and equalized signals at low (5 dB) and high input SNRs (15 dB) obtained by using a two-stage training method (see note S1 and fig. S1) for the hardware implementation of the proposed NN models on memristor crossbar arrays. The achieved signal-to-noise-and-distortion ratios (SNDRs) of 17.05 and 21.1 dB at 5- and 15-dB input SNRs (with additional results at other SNRs shown in fig. S2) indicate enhanced adaptability compared to traditional MMSE-based channel equalizers. The achieved SNDR versus SNR for signals with different carrier frequencies (see fig. S3) demonstrates that the NN-based channel equalizer not only compensates for channel distortion but also attenuates the in-band noise. This advantage contrasts with traditional equalization approaches, which correct signal attenuation and distortion at the cost of amplifying in-band noise. Experimental results (Fig. 2 and note S1) show that the proposed channel equalizer implemented with memristor crossbar arrays effectively reduces amplitude and phase distortions, compensates for channel-induced fading, and mitigates the effects of AWGN.

### Analog filter and modulation-based signal router

**Fig. 3.**
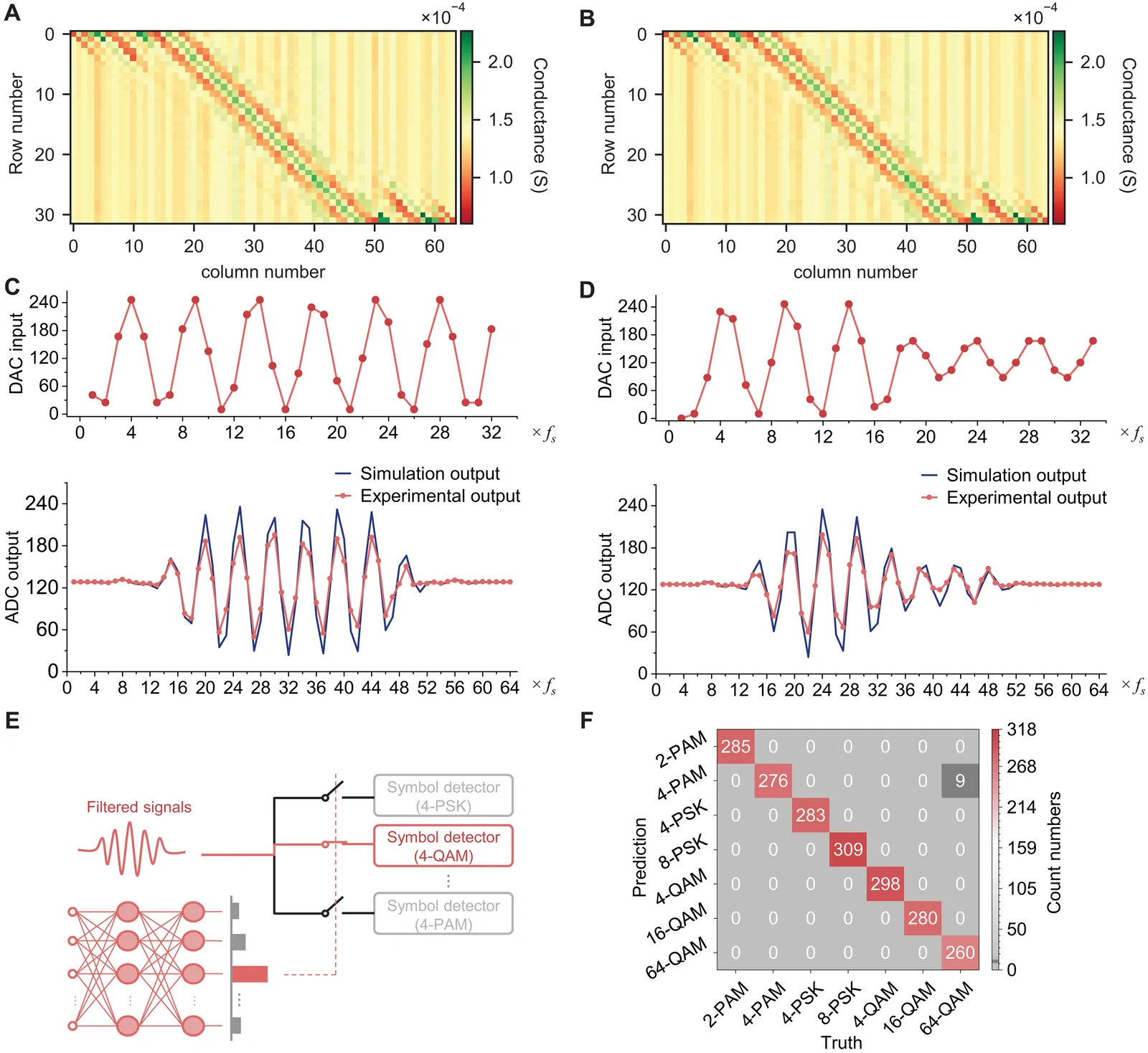
Analog FIR filter and NN-based signal router. (**A**) Target conductance map of the memristor crossbar array implementing a 32-point FIR filter, where the filter coefficients are arranged in a Toeplitz matrix structure. (**B**) Experimental conductance map of the FIR filter matrix after programming. (**C** and **D**) Representative FIR filtering results for different modulation schemes: 4-QAM and 4-PAM. The first row shows the input signals applied to the FIR filter; the second row compares simulated filter outputs with experimental hardware measurements. (**E**) NN-based signal router implemented using a multilayer perceptron (MLP) across multiple memristor crossbar arrays for modulation classification, directing the filtered signal to the corresponding symbol-detection NN. (**F**) Modulation classification performance for signals modulated using seven different schemes: 2-PAM, 4-PAM, 4-QAM, 16-QAM, 64-QAM, 4-PSK, and 8-PSK. The confusion matrix shows an average classification accuracy of 99.55% over 2000 test samples.

Filtering a signal that passes within a specific frequency band to block unwanted frequencies is a key function of a wireless receiver before the equalized signals can be used for symbol detection. Because an FIR filter can be reformulated as a convolution operation with a Toeplitz matrix, it is mathematically equivalent to a convolution layer with Toeplitz-structured weights (see Materials and Methods and fig. S4). Accordingly, we implement the analog filter using analog VMM in a memristor crossbar array, where the equalized signals are applied as inputs. Figure 3A illustrates the target conductance map of the memristor crossbar array when mapping a 32 by 64 FIR matrix to the conductance values of memristor devices in crossbar array (see Materials and Methods). Figure 3B shows the measured conductance map of the memristor crossbar array after programming the target conductance values into a memristor crossbar array. Figure 3 (C and D) displays the input signals before filtering and compares the theoretical and experimental outputs for two modulation types, a four-level quadrature amplitude modulation (4-QAM) and a four-level pulse amplitude modulation (4-PAM). Results for additional modulation types are shown in fig. S5. The MMSE of the filtered outputs across six modulation schemes remains within 5% (see note S2 and fig. S6). The low MMSE between theoretical and hardware outputs confirms the accuracy and adaptability of the FIR filter implementation. The analog FIR filter effectively suppresses unwanted frequencies and isolates the desired signal components, enhancing overall signal quality for symbol detection.

To support symbol detection across multiple modulation schemes, we design a modulation classification NN that functions as a dynamic signal router, as shown in Fig. 3E, directing filtered analog signals to corresponding NN-based symbol detectors according to their modulation schemes. This approach eliminates the need for multiple fixed demodulation chains while enhancing system flexibility and efficiency. We collected filtered signals modulated using seven widely used modulation schemes and use an MLP model for modulation classification (see note S3). The MLP is trained offline and mapped onto multiple memristor crossbar arrays for efficient inference. The performance of the modulation classification is shown in Fig. 3F as a confusion matrix, where the dominant diagonal elements indicate a high classification accuracy of 99.55% over 2000 testing samples. This high accuracy highlights the robustness of the MLP in reliably distinguishing multiple modulation types.

### NN-based symbol detector for data decoding

In traditional wireless receivers processing high-frequency signals, the local oscillator used in mixing introduces phase noise, frequency offset, and I/Q imbalance, all of which degrade signal integrity. These impairments increase system complexity and require additional compensation techniques to maintain reliable communication. To address these challenges, we use NNs to directly detect symbols from high-frequency signals, eliminating the need for traditional frequency down-conversion (*31*–*35*). Instead of relying on I/Q demodulation, our proposed NN-based symbol detector learns to map raw high-frequency waveforms to symbols, inherently compensating for distortions, enhancing robustness, and reducing receiver complexity. The NN-based symbol detector for each modulation scheme follows a CNN-MLP architecture (see Materials and Methods). The CNN layers extract initial phase information or phase differences associated with various modulation symbols, which is critical for accurately distinguishing symbols in phase-sensitive modulation schemes such as QAM and PSK. After extracting the relevant phase features, the MLP layers perform the final classification step, mapping the features to discrete symbol probabilities.

**Fig. 4.**
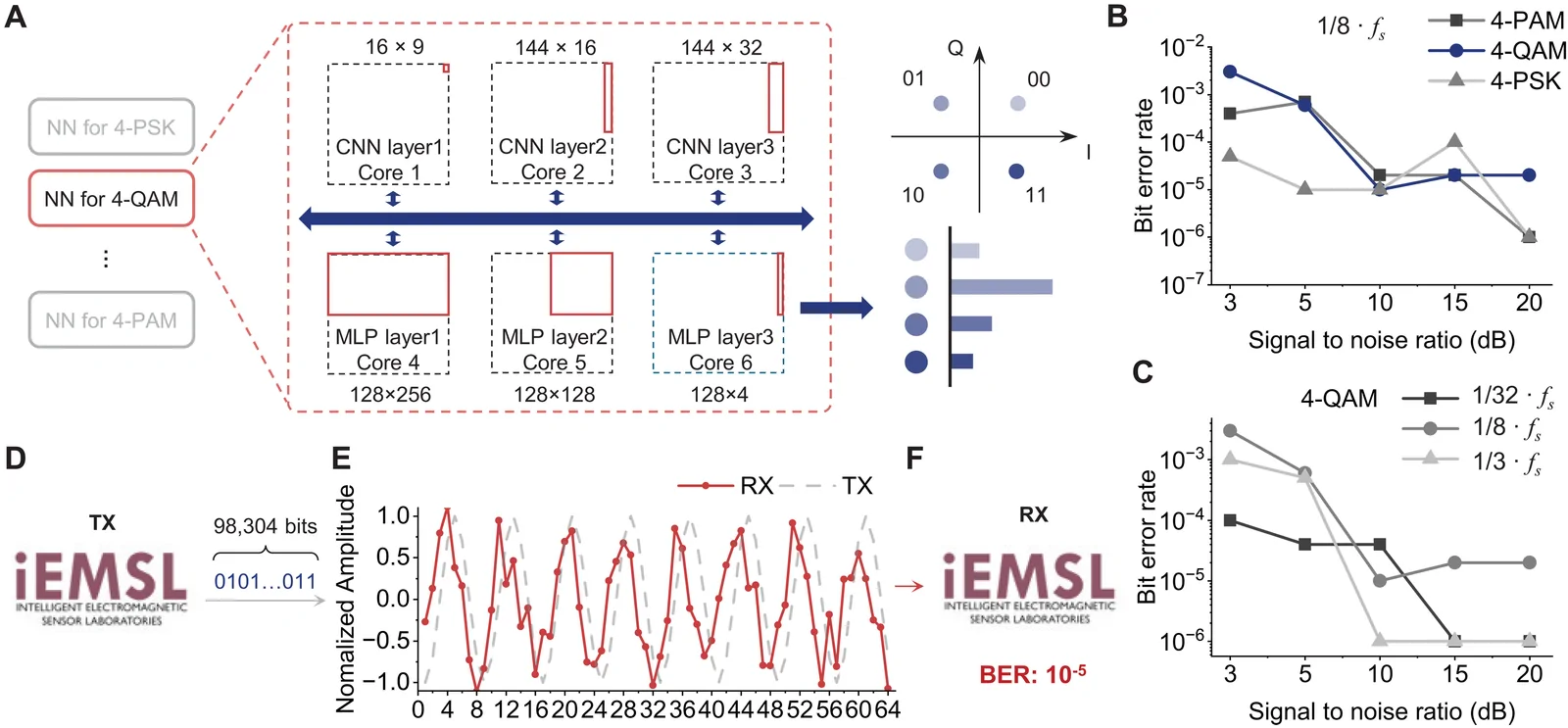
The NN-based symbol detector. (**A**) The symbol detector consists of three CNN layers and three MLP layers. The NN weights trained offline are mapped to different memristive computing cores for efficient inference. In the final MLP layer, the highest output current from the columns determines the symbol prediction for the input signal vector, corresponding to a symbol in the constellation map, with 4-QAM as an example. (**B**) The BER across different SNR after decoding 10^7^ to 10^8^ randomly generated symbols from analog signals with the same carrier frequency and modulated by different methods: 4-PAM, 4-QAM, and 4-level phase shift keying (4-PSK), with their corresponding symbol detector. (**C**) The BER across different SNR after decoding 10^7^ to 10^8^ randomly generated symbols from analog signals modulated by the same 4-QAM modulation method with different carrier frequencies ranging from DC to $1/2\cdot f_{s}$. (**D**) The image is part of the data (98,304 bits representing pixel values) modulated using 4-QAM for the experiments. (**E**) Part of the distorted analog signals received by the receiver (RX), carry the image data. (**F**) The decoded image from the analog wireless receiver within the memristive SoC achieves a BER of approximately 10^−5^.

As shown in Fig. 4A, an exemplar 4-QAM symbol detector is mapped onto multiple memristor crossbar arrays for efficient inference (see note S4 and fig. S7), and the final outputs of the MLP layers represent a probability distribution over possible symbols where the decoded symbol corresponds to the highest probability. To evaluate the effectiveness of the symbol detector for analog signals modulated with various schemes generated from a simulated transmitter (note S5 and fig. S8), we analyze the BER versus SNR performance when decoding digital data (see Materials and Methods) using analog signals modulated by three schemes: 4-PAM, 4-QAM, and 4-PSK, as shown in Fig. 4B. The results indicate that as SNR increases, BER decreases, with a BER of 10^−6^ at 20-dB SNR, demonstrating decoding accuracy under higher-quality signal conditions. The network exhibits robust performance across different modulation types, with particularly higher accuracies for phase-modulated signals, due to the phase detection capability of CNN layers. To demonstrate the adaptability of the proposed wireless receiver, we further demonstrate its operation over a wide frequency range, from direct current (DC) to $f_{s}/2$. To evaluate this capability, we test the proposed wireless receiver using analog signals modulated by 4-QAM with different carrier frequencies (1/32 $f_{s}$, 1/8 $f_{s}$, and 1/3 $f_{s}$). As shown in Fig. 4C, the system maintains a consistent BER across these carrier frequencies under varying SNRs, thus, validating the ability of our NN-based approach to directly decode symbols from analog waveforms at different frequency bands. This consistent BER performance proves that the NN extracts modulation features that are invariant to carrier frequency shifts, effectively eliminating the need for mixers or frequency down-conversion.

Figure 4D displays an image used to validate the proposed wireless receiver. In the experiment, the image is first digitized into a bitstream (98,304 bits), which is then modulated into symbols using 4-QAM. After transmission through the simulated channel, the received analog signal (an example is shown in Fig. 4E) is processed by the channel equalizer, routed by the signal router and decoded by the symbol detector (hardware workflow is detailed in note S6 and fig. S9). The receiver successfully decodes the received symbols, enabling image reconstruction from the detected symbols, as shown in Fig. 4F. The system achieves a BER of 10^−5^ for image data decoding, confirming the high accuracy and reliability of the proposed approach.

## DISCUSSION

We have demonstrated a proof-of-concept analog wireless receiver in which multiple NNs designed for signal processing are distributed across multiple memristor crossbar arrays within a memristive SoC. The proposed receiver outperforms both traditional signal processing algorithms (*36*, *37*) and digital hardware platforms (*38*–*41*). At the algorithm level, the modular NN-based signal processing offers high adaptability, robustness, and scalability, while it avoids explicit channel estimation at the cost of additional training phase and increased complexity (see table S1). These costs are mitigated by implementing NNs with AIMC hardware based on memristor crossbar arrays. As summarized in tables S2 and S3, the proposed analog wireless receiver achieves energy efficiency of 21.3 TOPS/W and an estimated communication throughput of 3.968 Gb/s. Both metrics are estimated from experimentally measured data and account for contributions from peripheral circuits, including transimpedance amplifiers (TIAs), digital-to-analog converters (DACs), ADCs, and on-chip communication (note S7 and fig. S10). These values exceed those of established digital processing platforms such as DSPs (*38*, *39*) (0.02 to 2.6 TOPS/W), field-programmable gate arrays (*40*) (FPGAs; 1.35 TOPS/W), and tensor processing unit (*41*) (0.8 TOPS/W). Compared with existing AIMC-based signal processing approaches, our work represents the first experimental implementation of NN-based data decoding using memristor crossbar arrays (see note S8 and table S4). Previous studies typically rely on small crossbar arrays [for example, 32 by 64 (*42*) or 64 by 64 (*24*)] to validate individual functions and depend on simulations for system-level evaluation. By contrast, our receiver uses multiple on-chip 248 by 256 memristor crossbar arrays to experimentally demonstrate complete data decoding across multiple modulation schemes and carrier frequencies.

However, several challenges remain for the proposed NN-based wireless receiver. The current memristor crossbar arrays within the SoC support only 8-bit analog precision and are affected by peripheral nonidealities, including IR drop, signal distortion, and ADC noise. Consequently, the present experimental demonstration is restricted to data decoding over SNRs ranging from 3 to 20 dB using 4-QAM, 4-PAM, and 4-PSK modulation schemes. Although simulations demonstrate that the proposed receiver requires only a small number of retraining steps to adapt to changing channel conditions and can be extended to higher-order modulation schemes, including 16-, 64-, 128-, and 256-QAM (see note S9 and tables S5 and S6), implementing these schemes on the current memristive SoC remains challenging. Higher-order constellations exhibit smaller decision margins, rendering analog signal processing increasingly sensitive to hardware nonidealities, and achieving comparably low BER with higher-order modulation requires approximately doubling both the depth and width of the equalizer and symbol detector NN modules. Therefore, further improvements to the memristive hardware, including scaling to larger memristor crossbar arrays to accommodate wider NN architectures and reducing hardware nonidealities, are required.

Another limitation concerns the energy and area efficiency overhead arising from the requirement for DACs and ADCs in the memristive SoC used to demonstrate the proposed analog wireless receiver. Currently, DACs are required to convert intermediate results between NN layers into analog voltages applied to the memristor crossbar arrays, while ADCs are necessary to convert the resulting outputs back into digital data for on-chip communication between multiple memristor crossbar arrays (note S6), since fully analog on-chip communication remains challenging even for state-of-the-art AIMC hardware (*18*, *21*, *43*, *44*). Consequently, peripheral circuits, including TIAs, DACs, and ADCs, still dominate the overall power consumption and chip area, each exceeding 50% of the respective totals (note S7). This limitation could be addressed through advances in sample-and-hold circuits and fully analog on-chip communication, which would eliminate the need for DACs and ADCs. In addition, replacing the activation functions between NN layers with analog alternatives, such as analog sigmoid circuits (*45*) and rectified linear unit (ReLU) circuits (*17*, *46*), would reduce the number of TIAs required, which would then only be needed between distinct NN modules. Furthermore, future memristive SoCs could integrate edge learning capabilities (*47*) to enable on-chip training of NNs, which would also enhance both the adaptability and energy efficiency of the NN-based wireless receiver.

In summary, we present an analog wireless receiver architecture in which modular NNs directly process continuous-time RF signals for data decoding, and demonstrate the approach with AIMC hardware based on memristor crossbar arrays. The channel equalizer effectively mitigates distortions in analog signals across varying input SNRs. The analog filter successfully suppresses unwanted frequencies, while the signal router leveraging modulation classification achieves an accuracy of 99.55%. The NN-based symbol detector decodes data with a BER of approximately 10^−6^ across multiple modulation schemes and carrier frequencies, highlighting the adaptability of this architecture in practical applications. By integrating multiple NNs trained for RF signal processing, our system eliminates the mixer and reduces front-end complexity and lowers overall power consumption relative to traditional superheterodyne or direct-conversion receivers. These results underscore the potential of our approach for future wireless communication networks with high adaptability, energy efficiency, and reduced latency.

## MATERIALS AND METHODS

### NN designs for signal processing and data decoding

The network architecture for channel equalization consists of three CNN blocks and two MLP layers. The input of the network is 64-point analog samples of the distorted signals. Each CNN block comprises a convolutional layer with 3 by 3 kernels, a ReLU activation layer, and an average pooling layer with 2 by 2 kernels. The MLP layers also use the ReLU activation function. Before the NN channel equalizer, an encoder is used for channel feature extraction. This encoder consists of four CNN blocks, each containing a convolutional layer with 3 by 3 kernels, a ReLU activation layer, and a max-pooling layer with 2 by 2 kernels. The input to the encoder consists of 64-point analog signals sampled from the pilot symbols. Normalization layers are not included in the channel equalizer NN or the encoder since signal processing is highly sensitive to amplitude variations and amplitude ratios across different modulation types. The 64 outputs from the channel equalizer NN correspond to the equalized signals.

The signal router is implemented as an MLP with two hidden layers, each containing 128 neurons, for modulation classification. Each layer uses the ReLU activation function. The input to the network consists of a 64-point analog signal sampled from the filtered output of a VMM-based filter. The network has seven output neurons, each corresponding to one of the seven modulation schemes for data modulation (see details in note S3).

The network architecture for symbol detection is tailored to signals modulated by each modulation scheme and consists of three CNN blocks followed by three MLP layers. Each CNN block comprises a convolutional layer with 3 by 3 kernels, a ReLU activation layer, and an average pooling layer with 2 by 2 kernels. The MLP layers contain 128, 128, and 64 hidden neurons, respectively, with ReLU as the activation function for each layer. The input to the symbol detector consists of a filtered signal routed by the signal router, while each output corresponds to a symbol in the respective modulation scheme (see details in note S4). The details of key parameters of all proposed NN modules are shown in note S10 and tables S7 to S10.

### Bitstream generation and channel simulation

The distorted analog signal used to demonstrate the proposed wireless receiver within the memristive SoC is generated using a simulated transmitter and transmission channel (see note S5). We used software to simulate the data generation and transmission processes. In this simulation, a fixed pilot sequence (“0000 0101 1100 1111”) is repeated according to the modulation scheme to form 64 pilot symbols, followed by a 10^7^-bit random test sequence. Both pilot and test data are modulated, upconverted, and passed through a flat Rayleigh fading channel. The first 4096 samples (64 symbols) serve as pilots for channel feature extraction in the equalizer, while the remaining samples are processed as test data for distortion mitigation (see note S5).

For the image-based demonstration shown in Fig. 4 (D and E), we first convert the red, green, and blue (RGB) pixel values into a bitstream. This bitstream is then modulated using 4-QAM, followed by the same simulated transmission process described above. Last, the received distorted signals are processed by the proposed wireless receiver within the memristive SoC.

### Analog FIR filter

In a traditional FIR filter, each output sample is computed through a linear convolution of the input signal with a set of fixed filter coefficients. However, the use of static multiplier coefficients limits its ability to adapt to time-varying spectral environments. To efficiently implement FIR filtering in a memristor crossbar array, we restructure the convolution operation as a VMM using a Toeplitz matrix formulation. By reformulating this operation in matrix form, the input signal is embedded into a Toeplitz matrix, where each row represents a time-shifted instance of the input sequence, and the filter coefficients are arranged as a column vector. We map the input vectors to voltages applied to the rows of the memristor crossbar array, while the filter coefficients are programmed as the conductance of the memristor devices in the array. Under this mapping, the convolution operation can be expressed as

$$\begin{array}{cc} I_{\text{out}} & =\left[v_{0},v_{1},\cdots ,v_{N-1}\right]{\left[\begin{array}{ccccccc} G_{b0} & G_{b1} & \ldots & G_{bN-1} & G_{bN} & \;\;\;\;0\;\;\;\;\;\;\;\ldots & 0\\ 0 & G_{b0} & \;\;G_{b1} & \ldots & \;\;\;G_{bN-1}\; & \;\;\;G_{bN}\;\;\ddots & \vdots \\ \vdots & \ddots & \ddots & \ddots & & \ddots \;\;\;\;\;\;\;\;\;\ddots & 0\\ 0 & \cdots & 0 & G_{b0} & G_{b1} & \ldots \;G_{bN-1} & G_{bN} \end{array}\right]}_{N\times 2N}\\ & =\left[v_{0}\cdot G_{b0},\;v_{0}\cdot G_{b1}+v_{1}\cdot G_{b0},\;\cdots ,\;v_{N-1}\cdot G_{\mathrm{bN}}\right]\\ & =\left[v_{0},v_{1},\cdots ,v_{N-1}\right]*\left[G_{b0},G_{b1},\cdots ,G_{\mathrm{bN}}\right] \end{array}$$

where $G_{\mathrm{bi}},i=0,1,2,\cdots ,N$ represent the conductance values mapped from the FIR filter coefficients. Since the input vector has a size of (1×N) and the filter Toeplitz matrix has a size of (N×2N), the order of the FIR filter is N, with N+1 coefficients. Consequently, the VMM implementation of the FIR filter effectively performs the convolution of the input signal with the impulse response of the filter.

### Analog VMMs with memristor crossbar arrays in a SoC

The most energy- and time-intensive operations in NNs are VMMs. In a memristor crossbar array, VMMs are executed in a single physical step by exploiting Ohm’s law and Kirchhoff’s current law (*20*, *44*, *48*). The input vector is applied as analog voltages to the rows of the crossbar array, where it is multiplied by the memristor conductance values representing the matrix. The resulting column currents inherently perform the weighted summation, yielding the output current vector without sequential multiply-accumulate operations. In our memristive SoC, input vectors are stored in on-chip memory and converted to analog voltages by dedicated on-chip DACs within each memristive computing core. The matrix values are first mapped to approximately 255 conductance states of memristor devices, spanning a range of 2.55 to 650 μS, using a hardware-aware mapping scheme (*21*). The mapped conductance matrix is then programmed to the memristor crossbar array, which integrates Ta/HfO_2_-based memristors (*19*, *49*), using the optimized fine-tuning method (*50*, *51*). The resulting currents are converted to voltages by on-chip TIAs and digitized by on-chip ADC within each memristive computing core. The VMMs across multiple memristive computing cores within the SoC are managed by a two-stage RISC-V CPU (*21*), which is connected to a high-bandwidth on-chip advanced extensible interface data bus for data movement in the digital domain. In addition, the system includes a scatter-gather direct memory access engine to enable efficient on-chip core-to-core communication within the chip.

## Supplementary Materials

### This PDF file includes:

Supplementary Notes 1 to 10 Figs. S1 to S10 Tables S1 to S10 References
