# Supplementary material for: Analog neural network–based wireless receiver
Source: Sci Adv. 2026 Sep 11;12(37):eaeg7153. doi: 10.1126/sciadv.aeg7153 (PMC13564820; doi:10.1126/sciadv.aeg7153)
Supplement: Supplementary file 1 [file sciadv.aeg7153_sm.pdf]

Science Advances
AAAS

Supplementary Materials for

Analog neural network–based wireless receiver

Chaoyi He *et al.*

Corresponding author: Qiangfei Xia, qxia@umass.edu; Linda Katehi, katehi@tamu.edu

Sci. Adv. **12**, eaeg7153 (2026)
DOI: 10.1126/sciadv.aeg7153

This PDF file includes:

Supplementary Notes 1 to 10
Figs. S1 to S10
Tables S1 to S10
References

Supplementary Notes

Supplementary Note 1: Two-stage training and performance of NN-based channel equalizer

The channel equalizer is designed to mitigate wireless channel distortions and noise using an NN-based approach that dynamically adapts to varying conditions. It comprises a dedicated encoder for channel feature extraction and following NN layers for signal equalization. The encoder processes pilot symbols embedded within the received signals, which serve as known references to characterize key channel impairments such as fading, phase distortion, and noise interference. It maps these pilot symbols into a compact feature representation that captures the channel state. This feature vector is then concatenated with the raw received signals and fed into the NN model for equalization. The NN model for signal equalization consists of three convolutional neural network (CNN)(*52*) layers followed by two multilayer perceptron (MLP)(*53*) layers. The training phase of the equalizer minimizes the error between the outputs of the NN model and the transmitted signals thus enabling the model to learn generalizable features for channel equalization. To address hardware imperfections when implementing the equalizer with memristor crossbar arrays, a two-stage training strategy is implemented as shown in Figure S1. In the first stage, the NN is initially trained, and the first three CNN layers are programmed onto one memristor crossbar array. In the second stage, the hardware outputs from these CNN layers are used to retrain the remaining MLP layers. This hardware-in-the-loop retraining allows the remaining MLP layers to adapt to and compensate for hardware-specific interferences, effectively mitigating performance degradation caused by hardware imperfections.

To evaluate the performance of the channel equalizer, we process analog signals modulated using 4-level quadrature amplitude modulation (4-QAM) with signal-to-noise ratios (SNR) of 3, 5, 10, 15, and 20 dB through a simulated transmission channel (see Supplementary Note 5). We present a comparison between the distorted and equalized signals at 5 dB and 15 dB SNR in the main text, while results for other SNR levels are provided in Figure S2.

We use the signal-to-noise and distortion ratio (SNDR) to evaluate signal quality by comparing the power of the fundamental signal to the combined power of noise and distortion. To compute SNDR, we follow these steps:

1. Acquire a time-domain signal, typically generated using a pure sine wave.
2. Perform frequency analysis via a Fourier Transform (FFT) to identify the fundamental frequency bin and measure the power of the fundamental frequency.
3. Sum the power in all other frequency bins, excluding the fundamental (and sometimes the DC component), to obtain the noise and distortion power.

4. Compute SNDR by dividing the fundamental signal power by the combined noise and distortion power and express the result in decibels (dB):

$$SNDR(dB) = 10\log_{10}\frac{P_{signal}}{P_{noise} + P_{distortion}}$$

This metric provides a quantitative assessment of signal integrity thus helping evaluate system performance in noisy and distorted environments. The output SNDR as a function of input SNR is shown in Figure S3. The equalizer consistently enhances SNDR compared to the distorted signals across most of the SNR range, outperforming it by more than 10 dB in some cases. This demonstrates that once the channel equalizer converges, it effectively mitigates channel impairments. Regardless of whether the signal is at low ($1/32 f_s$), middle ($1/8 f_s$), or high ($1/4 f_s$) frequencies, the equalizer consistently enhances SNDR compared to the distorted input, demonstrating frequency-independent performance. The consistent gain indicates that the network effectively learns time-domain features rather than frequency-specific patterns, allowing it to generalize across the entire supported spectrum without additional retraining. This property is critical for wideband or frequency-agile applications, where carrier frequency can shift dynamically. Overall, the results confirm the effectiveness of the equalizer in improving SNDR.

Supplementary Note 2: Performance of VMM-based finite impulse response (FIR) filter

We first demonstrate the VMM-based FIR filter by filtering a composite signal consisting of three sine waves at 1 MHz, 156 MHz, and 500 MHz; each sampled at 2 GHz. The FIR filter is designed to suppress the 1 MHz and 500 MHz components while preserving the 156 MHz signal. The experimental results are shown in Figure S4.

To demonstrate the adaptability of the VMM-based FIR filter for the proposed wireless receiver, we filter out unwanted frequencies of the analog signals modulated by 2-level and 4-level Pulse Amplitude Modulation (2-PAM and 4-PAM), 4-, 16-, and 64-level QAM (4-QAM, 16-QAM, 64-QAM), and 4- and 8-level Phase Shift Keying (4-PSK and 8-PSK). We present the FIR results for signals modulated by 4-QAM and 4-PAM in the main text, while results for other modulation schemes are provided in Figure S5.

We use Minimum Mean Square Error (MMSE)(*54*) as a quantitative evaluation of the filtered outputs from the memristive SoC. The MMSE is calculated as follows:

$$Error = \frac{1}{N}\frac{1}{D}\sum_{n=1}^{N}\sum_{d=1}^{D}(y_{id} - \hat{y}_{id})^2$$

where y_{id} is the theoretical output of the FIR filter of ith signal vector d^{th} output point and $\hat{y}_{id}$

is the experimental output of the FIR filter of ith signal vector d^{th} output point.

The MMSE of the filtered outputs for different modulation schemes are shown in Figure S6. The low MMSE across signals modulated by different modulation schemes demonstrated the adaptability of the VMM-based filter.

Supplementary Note 3: Training and testing data for modulation classification

The filtered outputs from the VMM-based filter serve as inputs to the NN for modulation classification. Analog signals modulated using seven different schemes (2-PAM, 4-PAM, 4-PSK, 8-PSK, 4-QAM, 16-QAM, and 64-QAM) are collected for NN training. To prevent bias in classification predictions, the signal lengths for different modulation schemes are adjusted to maintain approximately an equivalent size.

The training dataset consists of 1,000 samples per modulation scheme, totaling 7,000 sample vectors for the training process. A separate testing dataset is generated with approximately 280 sample vectors per modulation type (total 2000 testing sample vectors), following the same channel equalization and FIR filtering procedures as the training set. To compute the confusion matrix each entry represents the number of instances a received signal with a given modulation type is classified as a specific modulation type. Diagonal values indicate correctly classified signals, while off-diagonal values correspond to misclassifications. The observed 99.5% classification accuracy demonstrates the reliability of the NN-based modulation classification, with minimal misclassification across different modulation schemes. This high accuracy suggests that the trained NN effectively distinguishes between modulation types, ensuring a robust routing mechanism for the cognitive radio receiver. As a result, the filtered waveform is directed to the appropriate symbol detection NN which leads to high symbol detection performance.

Supplementary Note 4: NN design and performance of symbol detectors

Since signal amplitude is crucial for specific modulation schemes (e.g., QAM and PAM), a normalization layer is omitted in the symbol detection NN to preserve amplitude information. Additionally, since the initial phase of the waveform is critical for symbol detection in high-frequency modulation schemes (e.g., PSK and QAM), using a max pooling layer could lead to loss of key phase information, as the filtered waveform typically has lower values at the beginning. Instead, an average pooling layer with a small kernel is employed to reduce the CNN output vector dimension while retaining essential phase information within the latent vector. For the NN structures of symbol detectors across different modulation schemes, all layers remain the

same except for the output layer, which varies in the number of neurons. The number of output neurons is determined by:

$$\text{output neuron} = \log_2 \text{number of symbols}$$

We present the programmed conductance map of the symbol detector NNs for analog signals in Figure S7.

All NN models are trained using a quantization-aware training (QAT) method to enhance error tolerance and maintain performance under low-precision computation:

$$\mathcal{L}_{\text{total}}(y, \hat{y}, \theta) = \mathcal{L}_{\text{target loss}}(y, \hat{y}) + \sum_i \sum_j |\theta_i - c_j| \cdot \mathbb{1}\left(\theta_i \in [c_j - 0.05, c_j + 0.05)\right)$$

where y is the ground truth label, $\hat{y}$ is the prediction label, θ is the set of trainable parameters in the model and $\{c_j\} = \{-1, -0.95, -0.9, -0.85, -0.8, \cdots, 0.9, 0.95, 1\}$ is the set of quantization levels with a step of 0.1 or 0.05 depending on different jobs.

To calculate the BER with high confidence, we generate $10^7 \sim 10^8$ symbols for each modulation type during inference. The BER is calculated as:

$$\text{BER} = \frac{N_e}{N_t}$$

where N_e is the total number of erroneous bits (i.e., bits incorrectly detected). N_t is the total number of transmitted bits. For the image data decoding, we downsample the image to 64×128 pixels and convert it to uint8 data for the RGB channels. Each uint8 value is then transformed into an 8-bit binary vector thereby forming a complete binary stream. This stream is modulated using 4-QAM, resulting in 98,304 4-QAM symbols.

Supplementary Note 5: Simulated transmitter and Rayleigh fading channel

The whole data generation and transmission flow is described in Figure S8. In the simulation, the pilot data is a repeated sequence of "0000 0101 1100 1111". The number of repetitions varies depending on the modulation scheme, such that a total of 64 modulated symbols is collected. The test data consists of a randomly generated 10^7-bit stream of 0s and 1s with equal probability. The combined bitstream, pilot and test data, is divided into blocks of *n* bits, where *n* depends on the modulation type. Each block is then mapped to its corresponding symbol through modulation. The resulting symbols are subsequently upconverted with the carrier frequency in the time domain, using a sampling frequency identical to that of the receiver.

During the channel simulation, the transmitter samples are passed through a simulated wireless channel modeled as a flat Rayleigh fading channel since it accurately models wireless

communication in rich multipath environments where no dominant line-of-sight (LOS) component exists between the TX and RX. The channel is implemented using the "comm.RayleighChannel" function in MATLAB which incorporates multipath propagation, Doppler effects, and channel filtering. The simulated channel consists of six multipath components with delays of 0 ps, 200 ps, 800 ps, 1200 ps, 2300 ps and 3700 ps converted to seconds for simulation. The corresponding average path gains are 0 dB, -0.9 dB, -4.9 dB, -8 dB, -7.8 dB and -23.9 dB which represents the relative power levels of each multipath component. To simulate frequency variations due to motion, a maximum Doppler shift of 30 Hz is included. Additionally, channel filtering is enabled to apply the fading effect to the transmitted signal, and impulse and frequency response visualization is activated to analyze the channel characteristics. The simulation operates at a defined sampling rate (f_s), providing a comprehensive representation of real-world wireless environments with multipath fading and mobility effects.

In practical wireless scenarios, such as urban areas, indoor communications, and mobile environments, signals undergo multiple reflections, diffractions, and scatterings before reaching the RX. Rayleigh fading provides a statistical model to characterize these complex signal variations, rendering it widely useful for evaluating RX performance in non-line-of-sight (NLOS) conditions. Rayleigh fading introduces random amplitude variations due to constructive and destructive interference thus enabling rigorous testing of NN-based equalization, modulation classification, and symbol detection. By incorporating Doppler effects, the model also captures real-world mobility scenarios where frequency shifts occur due to relative motion between the TX and RX. Using a multipath Rayleigh fading model with different delay spreads and path gains evaluation of the adaptability of our system to dynamic, interference-prone wireless environments, ensuring realistic and generalizable performance assessments.

The resulting analog signals with simulated channel distortion are stored as digital data in the on-chip memory of the SoC for the demonstration of the proposed wireless receiver. The first 4096 samples (first 64 symbols) are designated as pilot samples and are concatenated with the corresponding pilot bit sequence to form the input to the channel feature extraction encoder within the channel equalizer. The remaining samples are treated as test data samples and are processed by the channel equalizer for distortion mitigation.

Supplementary Note 6: Hardware workflow of the NN-based wireless receiver

The hardware workflow of the NN-based wireless receiver is illustrated in Figure S9a. The distorted signals generated by the simulated transmitter and wireless channel (Supplementary Note 5) are stored in digital memory before being applied to the first module of the NN-based wireless receiver, the channel equalizer.

The architecture of one of the ten computing cores in the memristive SoC used for the wireless receiver demonstration is shown in Figure S9b. Each memristive computing core integrates a memristor crossbar array for analog VMMs in the CNN and MLP layers, together with essential peripheral circuits, including DACs, TIAs, ADCs, and control logic. Activation functions are also implemented within the same computing core using digital circuits. All VMMs across the CNN and MLP layers are executed in the memristor crossbar arrays of the computing cores, whereas the digital CPU is only used to manage digital data transfer between layers.

For the NN-based wireless receiver, the distorted signals stored in memory are first processed by the channel equalizer, which comprises three CNN layers and two MLP layers. The input data are converted into analog voltages within the memristive computing core and applied to the memristor crossbar arrays to perform analog VMMs for the convolution operations. The resulting output currents are subsequently converted into voltages by the TIAs and digitized by the ADC within the same core, after which the outputs are transferred to the next layer through the on-chip data bus. Following channel equalization, the equalized signals are stored in digital memory and processed by a VMM-based filter. The filtered signals are then applied to the signal router, which comprises two MLP layers. The classification output of the signal router is used by the digital CPU to determine the appropriate symbol detector to which the signals should be directed. The filtered signals are then routed to the corresponding symbol detector, comprising three CNN layers and three MLP layers, for symbol detection. Finally, the symbol detection results, represented as the classification outputs of the NNs, are transmitted to the digital CPU, which decodes the received bitstream.

Supplementary Note 7: Energy and area efficiency analysis

We estimate the peak energy and area efficiency of the memristive SoC used in the demonstration based on experimentally measured power consumption and chip area. The energy efficiency is defined as:

$$\text{Energy efficiency}=\frac{\text{Throughput}}{\text{Measured power}}$$

And the area efficiency is defined as:

$$\text{Area efficiency}=\frac{\text{Throughput}}{\text{Computing area}}$$

The throughput is estimated based on the full capability of the SoC as:

$$\text{Throughput}=\frac{N_{\text{operations}}}{t_{\text{operations}}}$$

As only one of the computing cores with 248 × 256 1T1R cells is active in the memristive SoC, and one VMM corresponds to a single dot product and one addition operation, each VMM requires 50 clock cycles at the operating frequency of 100 MHz. Accordingly, the throughput was calculated as

$$\text{Throughput}=\frac{248\times 256\times 2}{50\times\frac{1}{100\text{ MHz}}}=0.254\text{ TOPS}$$

To measure the average power consumption of the entire SoC, including the memristive computing cores and digital peripheral circuits, we executed VMMs within the computing cores 240,000 times and measured the average power. The average power consumption is 11.922 mW. Therefore, the energy efficiency of the SoC is calculated as:

$$\text{Energy efficiency}=\frac{0.254\text{ TOPS}}{11.922\text{ mW}}=21.3\text{ TOPS/W}$$

The area for the computing area, including one memristor crossbar array and peripheral circuits, such as TIAs, DACs, and ADCs, is 8.4 mm^2, Therefore, the area efficiency of the SoC is calculated as:

$$\text{Area efficiency}=\frac{0.254\text{ TOPS}}{8.4\ mm^2}=0.03\text{ TOPS}/mm^2$$

The energy and area efficiency can be further improved in future developments. As shown in the power and area breakdown of the memristive SoC in Figure S10, the overall power consumption and chip area are still dominated by peripheral circuits, including TIAs, DACs, and ADCs. Advances in sample-and-hold circuits and fully analog on-chip communication could eliminate the need for DACs and ADCs, thereby further improving the energy efficiency of the proposed wireless receiver. In addition, replacing the activation functions between NN layers with analog alternatives, such as analog sigmoid circuits(*45*) and ReLU circuits(*17*, *46*), would reduce the number of TIAs required.

Supplementary Note 8: Comparison with traditional and AIMC-based signal processing

Since the proposed analog wireless receiver, implemented with memristor crossbar arrays in a SoC, differs fundamentally from traditional receiver systems at both the algorithm and hardware levels, we provide a detailed comparison at both levels. Algorithm-level trade-offs are summarized in Table S1 and Table S2, while hardware-level performance comparisons are presented in Table S3 and Table S4.

Compared to traditional signal processing methods implemented on digital processing platforms*(36, 37)*, NN-based approaches offer greater adaptability to environmental changes and

enhanced robustness against channel variations, owing to the inherent learning capabilities of NN models. Specifically, NN-based channel equalization does not require explicit channel estimation, as it directly learns the mapping between the received signals and transmitted symbols from training data, thereby bypassing the need for explicit mathematical channel modeling. In contrast, conventional equalizers depend on estimating the channel impulse response or frequency response, commonly referred to as channel state information (CSI), and subsequently inverting or compensating for the channel effects using signal processing techniques. This approach is highly sensitive to inaccuracies in CSI, particularly under fast-fading or nonlinear channel conditions. However, the benefits of NNs are accompanied by trade-offs, including increased computational complexity and the necessity for additional training, as the NNs need to learn relevant signal features and distortions from the training signal dataset.

While prior works have implemented NN-based signal processing on digital platforms such as GPUs(*9, 55*) and FPGAs(*56*), most existing NN receivers are structured as a monolithic deep network that operates on baseband signals following a conventional mixer/LO front-end. Adapting such a network to new channel environments necessitates retraining the entire model, which requires thousands of steps, as shown in Table S2, and incurs substantial time and energy costs. In contrast, the proposed modular architecture directly processes high-frequency RF signals and can be retrained to adapt to changing channel environments in orders of magnitude fewer steps due to the modular design as reported in Table S2.

Since the memristive SoC used to demonstrate the proposed receiver is at the proof-of-feasibility stage, its computational capability remains limited compared to digital signal processor (DSP) and general-purpose platforms such as field programmable gate array (FPGA) and tensor processing unit (TPU). As a result, a direct comparison of a complete receiver system is challenging. Therefore, we focus on comparing communication throughput, a metric related to signal processing capability, and energy efficiency related to power consumption. The energy efficiencies of the compared systems are from the respective references, all evaluated under 8-bit operations for consistency. The energy efficiency of the proposed system is estimated based on measured data(*21*).

The communication throughput is estimated under the assumption that our system can be extended to an equivalent orthogonal frequency-division multiplexing (OFDM) system with 248 subcarriers, employing 256-QAM modulation. Under these conditions, the theoretical communication throughput is based on the equation:

$$\text{Communication throughput} = N_{\text{subcarriers}} \times \log_2 M \times \frac{N_{\text{SpatialStreams}}}{(t_{\text{DataInterval}} + t_{\text{GuardInterval}})}$$

where $N_{\mathrm{subcarriers}}$ is the number of subcarriers used for transmitting modulated data, $\log_2 M$ represents the number of bits per symbol based on the modulation scheme, $N_{\mathrm{SpatialStreams}}$ denotes the number of unique multi-input multi-output (MIMO) data streams that can be transmitted in parallel. The denominator corresponds to the symbol interval, which is the sum of the data interval time $t_{\mathrm{DataInterval}}$ and the guard interval $t_{\mathrm{GuardInterval}}$, and defined by IEEE 802.11a/g standards(*57*). Based on the formulation, the estimated communication throughput for our system, a fully integrated memristive SoC that includes ten memristive computing cores, each of which features a 248 × 256 1T1R crossbar array, is:

$$248 \times 8 \times \frac{8}{4\mu s} = 3.968 \text{ Gbps}$$

For the digital systems, we estimate the communication throughput of DSP(*38*) under the same 256-subcarrier OFDM assumption, since this platform only implements a basic radix-2 fast Fourier transform computing cell. While the DSP(*38*) can theoretically achieve a communication throughput comparable to that of our system, it demonstrates lower energy efficiency. Similarly, another DSP implementation(*39*) exhibits both reduced communication throughput and lower energy efficiency relative to our system. Compared to digital computing platforms, like FPGA(*40*) and TPU(*41*), the proposed receiver implemented with memristor crossbar arrays demonstrates higher energy efficiency, albeit with lower communication throughput than TPUs. (*9*)(*10*)(*11*)Compared with prior AIMC hardware for wireless signal processing (Table S4), the proposed receiver is the first to leverage neural networks for data decoding, which necessitates multiple functional blocks, including equalization, filtering, and symbol detection. In contrast, previous works primarily focus on demodulation, which converts modulated waveforms into baseband symbol sequences and typically requires only a single memristor crossbar array to implement the inverse modulation matrix, or on task-specific detection, where traditional methods are implemented with memristor crossbar arrays to identify the presence of particular signals. Moreover, all prior demonstrations rely on conventional signal processing algorithms. The proposed receiver is evaluated using 248×256 memristor crossbar arrays within a fully integrated SoC, providing a substantially higher level of integration than prior wireless AIMC implementations. Importantly, the reported energy efficiency is derived from measured data on the integrated chip, whereas previously reported energy efficiencies were typically estimated at the crossbar-array level and did not account for the energy overhead of peripheral circuits.

Supplementary Note 9: Adaptability and scalability of the NN-based wireless receiver

The proposed NN-based wireless receiver can adapt to changing channel environments with minimal retraining due to the modular architecture. We take the Rayleigh fading channel as the

baseline and evaluate adaptability under progressively larger channel shifts, spanning both parametric drift within the same channel family and transitions to structurally distinct channel models. Starting from the baseline, we first introduce a mild distribution drift by increasing the Doppler frequency from 30 Hz to 40 Hz while applying only small perturbations to the multipath structure, with path delays varied by ±5% and path gains by ±0.5 dB. We then introduce a substantial distribution shift by increasing the Doppler frequency to 80 Hz and completely reshuffling both path delays and path gains, fundamentally altering the temporal and multipath characteristics of the channel. To further assess generalization across different propagation conditions, we also consider transitions to distinct channel models. The LoS scenario replaces the baseline Rayleigh fading channel with a Rician channel (K = 10 dB) and reduces the Doppler frequency to 5 Hz, introducing a dominant direct path and substantially more deterministic channel behavior. The NLoS scenario adopts the CDL-A model with 11 propagation paths and a Doppler frequency of 50 Hz, yielding richer multipath propagation, greater delay spread, and faster channel variations. The progression from baseline through channel drift and channel shift evaluates adaptability to increasing parametric variation within the same channel family, whereas the LoS and NLoS transitions evaluate adaptability to more fundamental changes in channel statistics and propagation mechanisms. The number of retraining steps required to achieve comparable equalizer MSE and BER performance across all scenarios is reported in Table S5. All experiments share the same signal parameters (M-QAM with $M = 4$, $N = 64$ samples/frame, $f_c \approx 187.5$ MHz, $f_s = 1$ GHz, SNR = 15 dB) and incorporate memristor crossbar arrays non-idealities from hardware characterization. As shown in Table S5, only a few hundred retraining steps are sufficient to restore the equalizer to baseline MSE performance and the symbol detector to baseline BER performance, corresponding to merely microseconds of training data at a 1 GHz sampling rate.

In the hardware demonstration of the proposed NN-based wireless receiver, signal processing was validated only for 4-QAM, 4-PAM, and 4-PSK modulation schemes within the memristive SoC, due to hardware non-idealities and the limited number of available memristor devices for NN weights. The proposed modular NN architecture can be extended to higher-order modulation schemes, including 16-, 64-, 128-, and 256-QAM, as demonstrated by the simulation results presented in Table S6. However, higher-order constellations exhibit smaller decision margins, rendering analog signal processing increasingly sensitive to hardware non-idealities. Furthermore, when evaluated using the baseline network size supporting the lower-order schemes (approximately 9×10^4 weights), 128- and 256-QAM achieved BER no lower than the 10^{-2} order. Attaining comparable low BER required approximately doubling both the depth and width of the equalizer and symbol detector NN modules, resulting in roughly 6× more NN weights, as detailed in Table S6, a scale that exceeds the capacity of the current memristive SoC. Accordingly, extending the proposed NN architecture to higher-order modulation in memristive

hardware necessitates two complementary directions for future development, scaling to larger memristor crossbar arrays to accommodate wider NN architectures, and improving hardware non-ideality characteristics to satisfy the tighter noise margins demanded by higher-order constellations.

Supplementary Note 10: Key parameters of all proposed NN modules

The NN-based wireless receiver comprises three modules: a signal router (modulation classifier), a channel equalizer (pilot encoder + equalizer branch), and a symbol detector. All three operate directly on 64-point analog samples. Normalization layers are deliberately omitted everywhere in the receiver, because modulation classification and symbol detection are highly sensitive to amplitude variations and to amplitude ratios across different modulation types.

Signal router (MLP)

The signal router is a fully connected MLP with two hidden layers of 128 neurons each (ReLU activations) that classifies the modulation type into seven classes (PAM2/4, PSK4/8, QAM4/16/64). Its input is a 64-point analog signal sampled from the filtered output of a VMM-based filter, and its seven output neurons each correspond to one of the seven modulation schemes (Supplementary Note 3). The module has 25,735 trainable parameters (101 KB) and is trained for 30 epochs with the Adam optimizer (initial lr = 0.001, cosine annealing), a batch size of 32, and an 80/20 stratified train/validation split.

Channel equalizer (pilot encoder + equalizer branch)

The channel equalizer pairs a pilot encoder for channel-feature extraction with an equalizer branch that outputs the equalized samples. The pilot encoder takes 64-point analog samples of the pilot symbols and consists of five 3×3 convolutional layers (each followed by ReLU and 2×2 max-pooling) and two FC layers, giving 21,020 parameters (82 KB). The equalizer branch takes the 64-point distorted signal and consists of three CNN blocks, each a 3×3 convolution, ReLU, and 2×2 average-pooling layer, followed by two FC layers (ReLU), giving 84,064 parameters (328 KB); its 64 outputs are the equalized signals. The full model has 105,084 parameters (410 KB) and is trained for 300 epochs with Adam (initial lr = 0.01, cosine annealing), a batch size of 256, and ~31,936 training samples. For crossbar deployment, the convolutional front-end is mapped onto the hardware array and kept frozen, while hardware-aware retraining fine-tunes the final FC layers to absorb device non-idealities.

Symbol detector (CNN + MLP)

The symbol detector is tailored to each modulation scheme and consists of three CNN blocks (3×3 convolution + ReLU + 2×2 average-pooling) followed by three FC layers with 128, 128, and 64 neurons (ReLU). It has 28,596 parameters (112 KB). The input is the filtered signal

routed by the signal router, and each output corresponds to a symbol in the respective modulation scheme (Supplementary Note 4). The network is trained for 50 epochs with Adam (initial lr = 0.001, cosine annealing), a batch size of 512, and ~80,000 training samples (~6 min wall-clock).

Summary

In software training, the full receiver totals 159,415 trainable parameters (router 25,735 + full equalizer 105,084 + detector 28,596). In the deployed configuration, where the equalizer's pilot encoder serves only for pilot-time feature extraction and the equalizer branch's convolutional front-end is mapped onto the crossbar, the receiver totals 138,395 parameters (router + equalizer-HW + detector). This compact footprint makes the receiver well-suited to hardware implementation in edge devices such as our memristive SoC.

Supplementary Figures

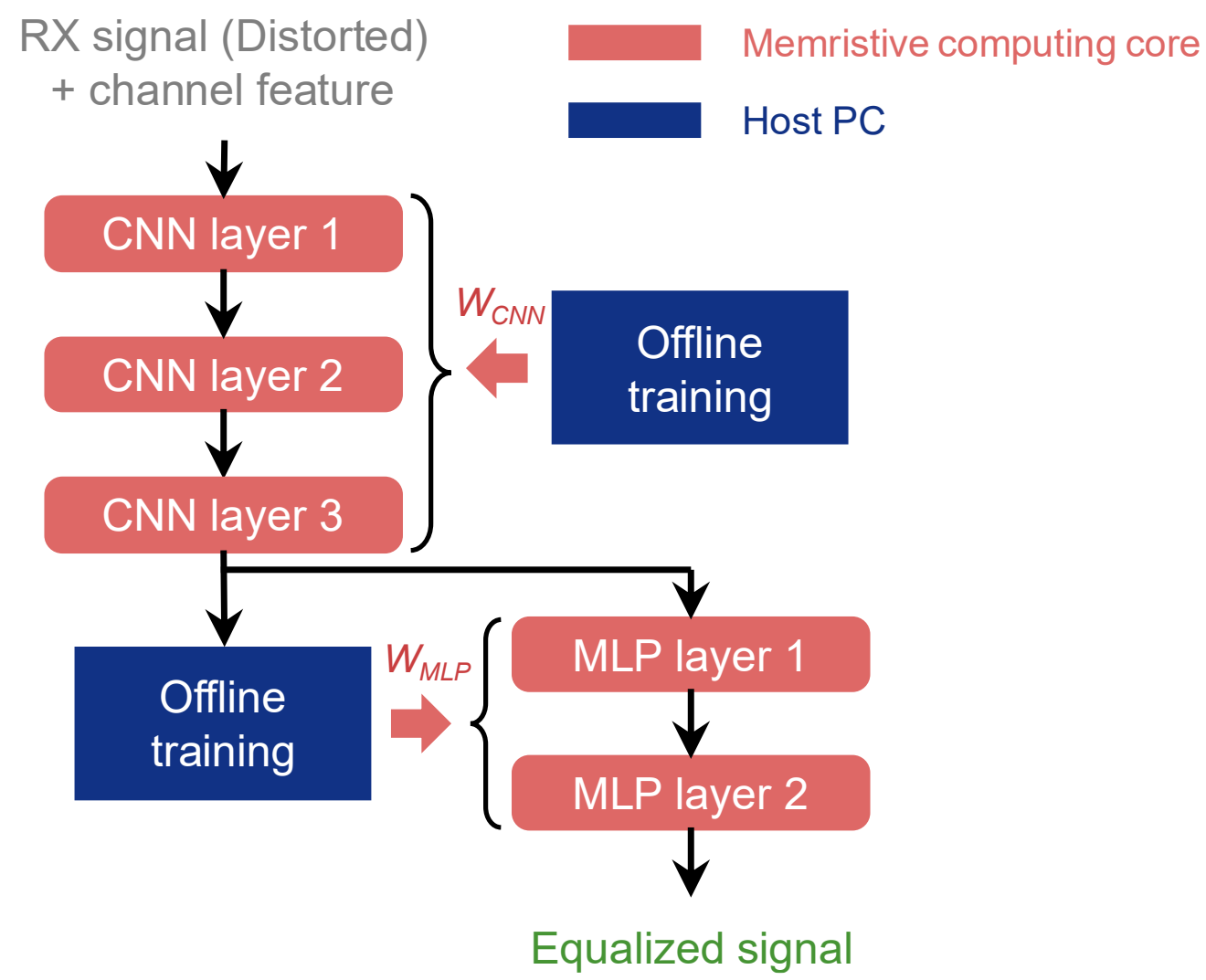

Figure S1 | Two-stage training of NN-based channel equalizer.

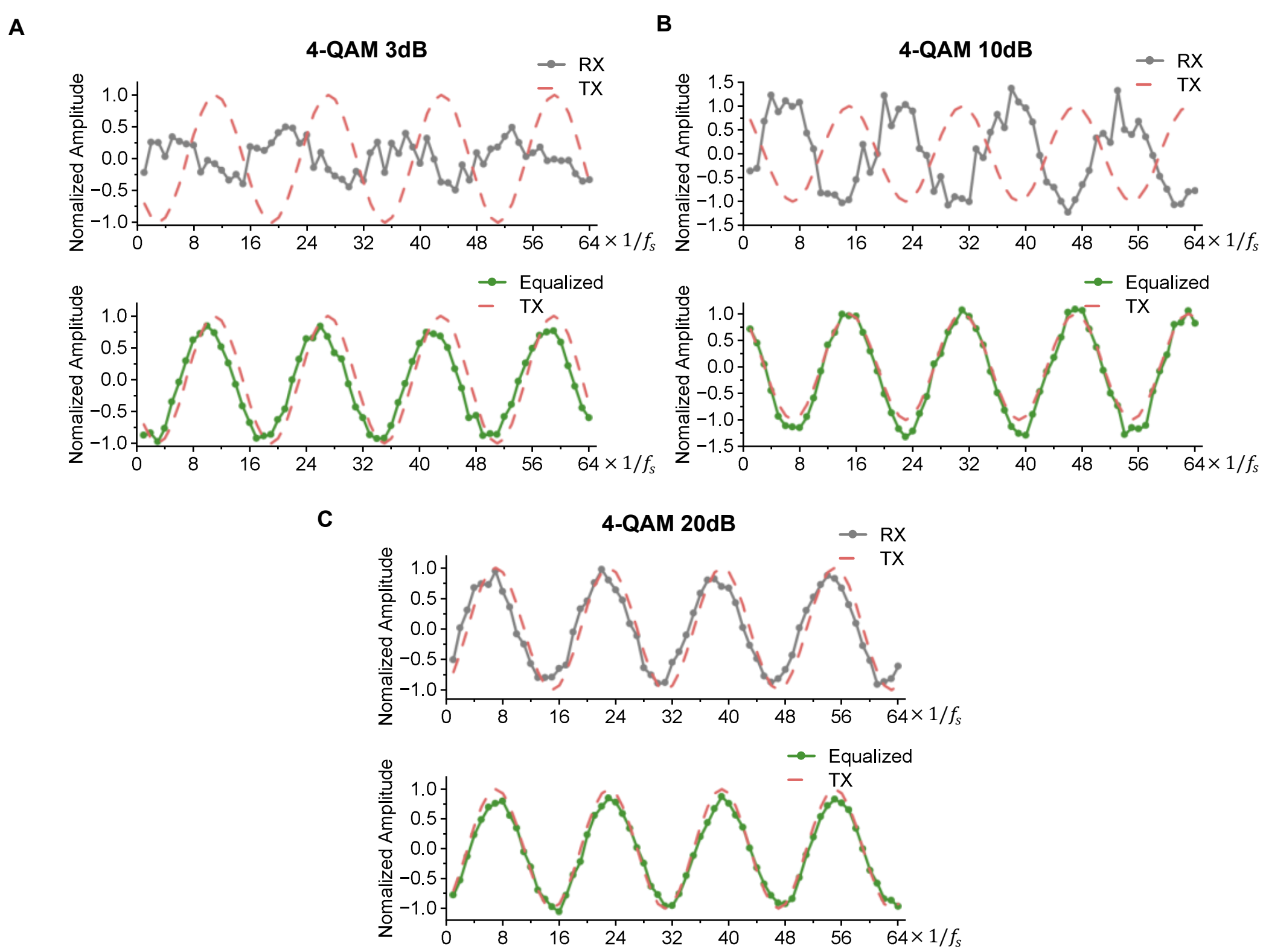

Figure S2 | The comparison between a part of the distorted signals received by the receiver (RX) and corresponding equalized signals, with the original signals from the transmitter (TX) serving as the reference.

A. SNR 3dB. **B.** SNR 10dB. **C.** SNR 20dB.

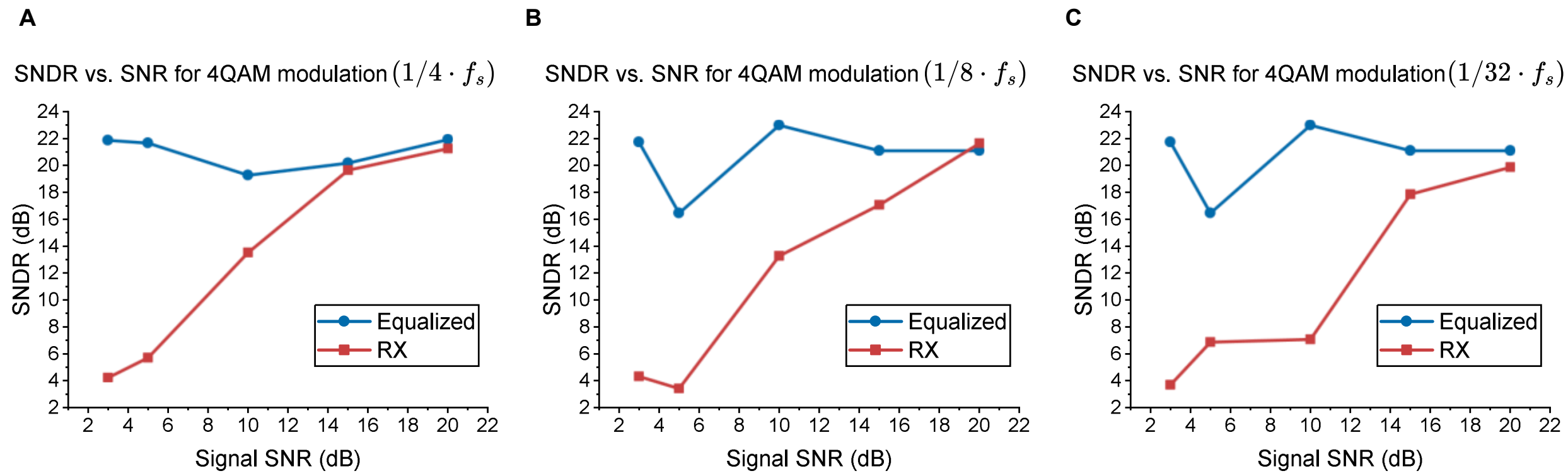

Figure S3 | The signal-to-noise and distortion ratio (SNDR) versus signal-to-noise ratios (SNR) of equalized (Blue) and received distorted (Red) signals modulated by 4-QAM across three different carrier frequencies.

A. High carrier frequency (1/4 f_s). **B.** Middle frequency (1/8 f_s). **C.** Low frequency (1/32 f_s).

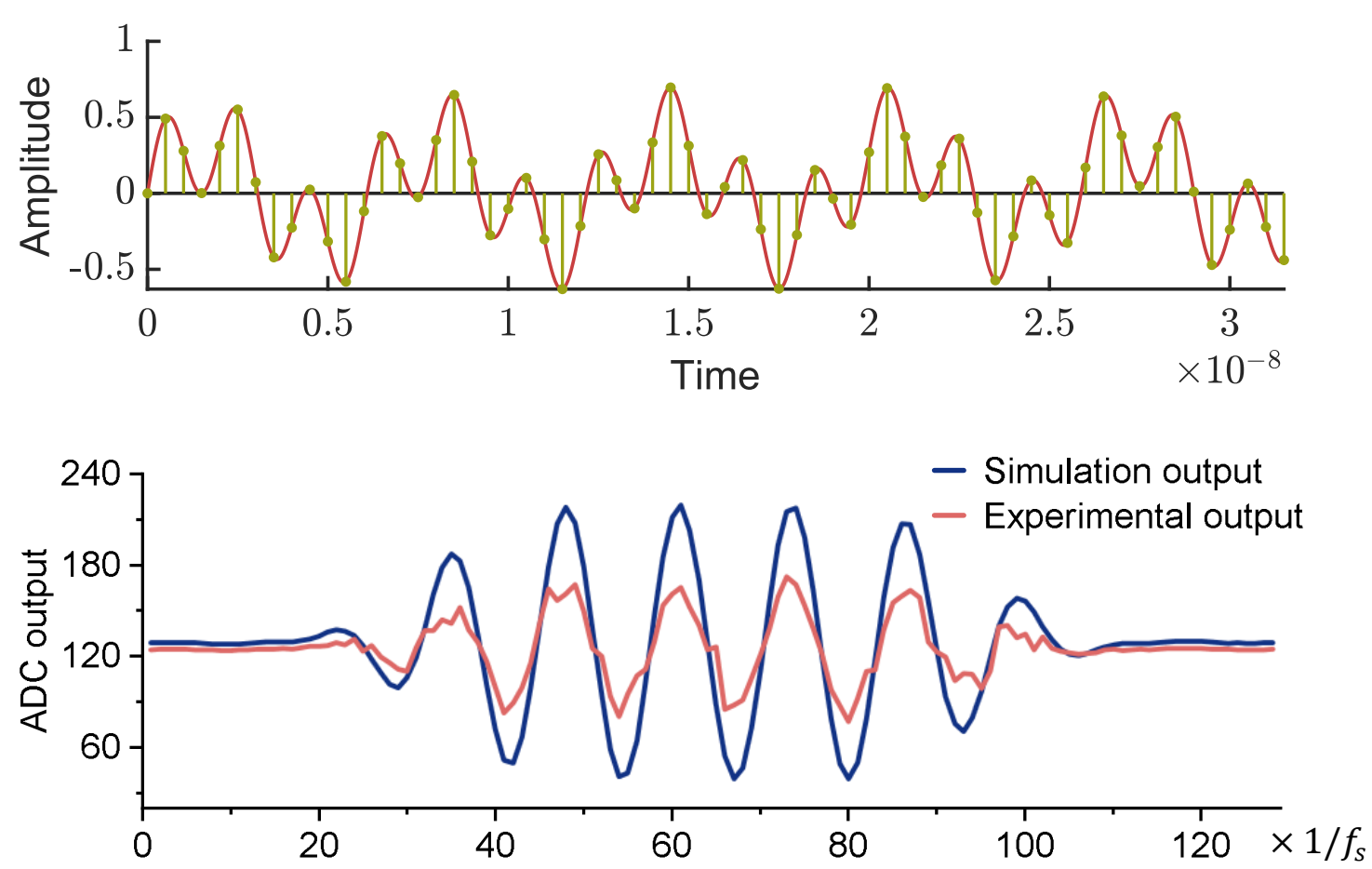

Figure S4 | Comparison of the original signals (a combination of three sine waves) and the filtered outputs from the VMM-based FIR filter.

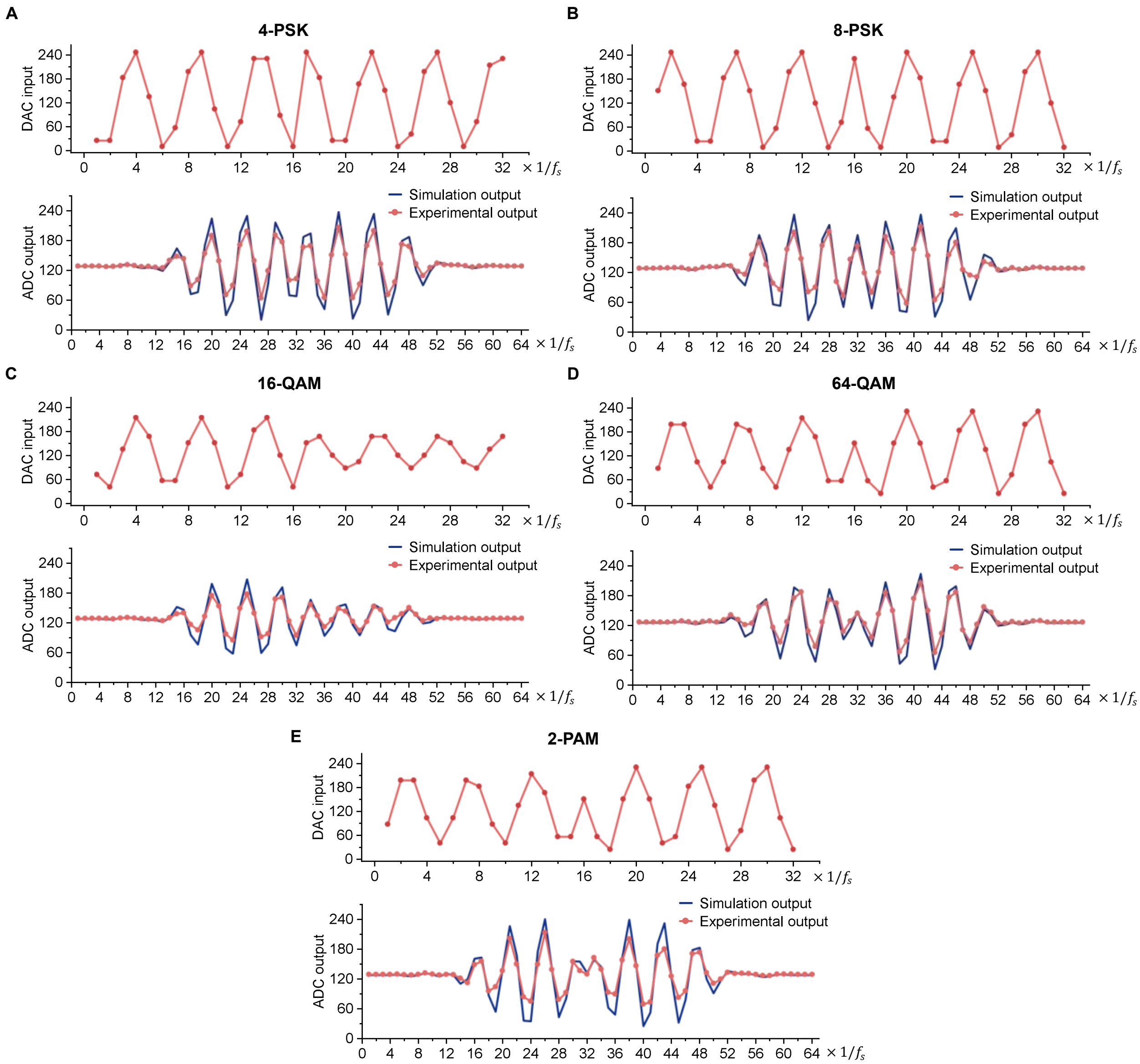

Figure S5 | Parts of the FIR filter results for different signal modulation schemes. **A.** 4-PSK. **B.** 4-PSK. **C.** 16-QAM. **D.** 64-QAM. **E.** 2-PAM.

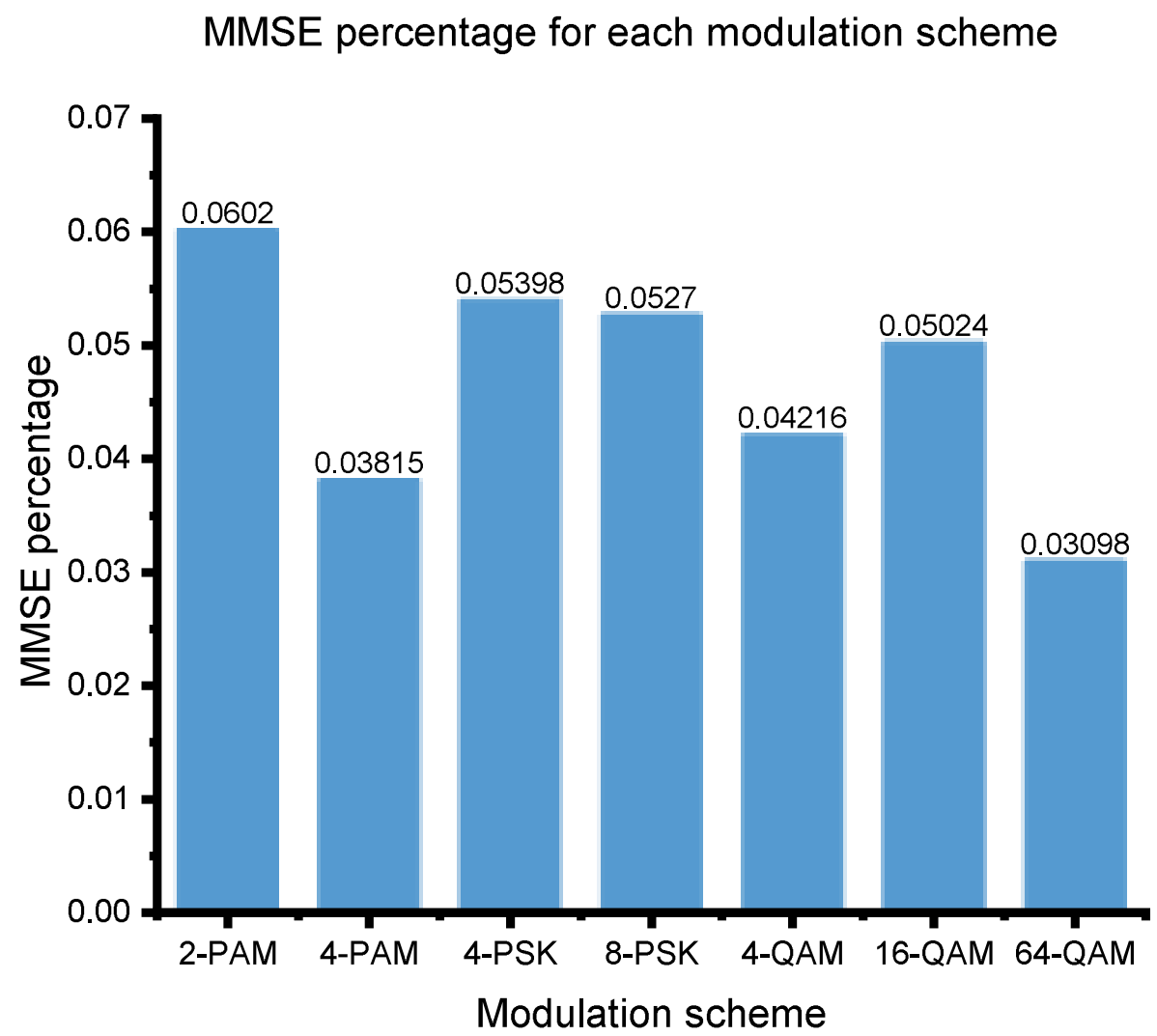

Figure S6 | The Minimum Mean Square Error (MMSE) of the filtered outputs across various modulation schemes after processing through the VMM-based FIR filter.

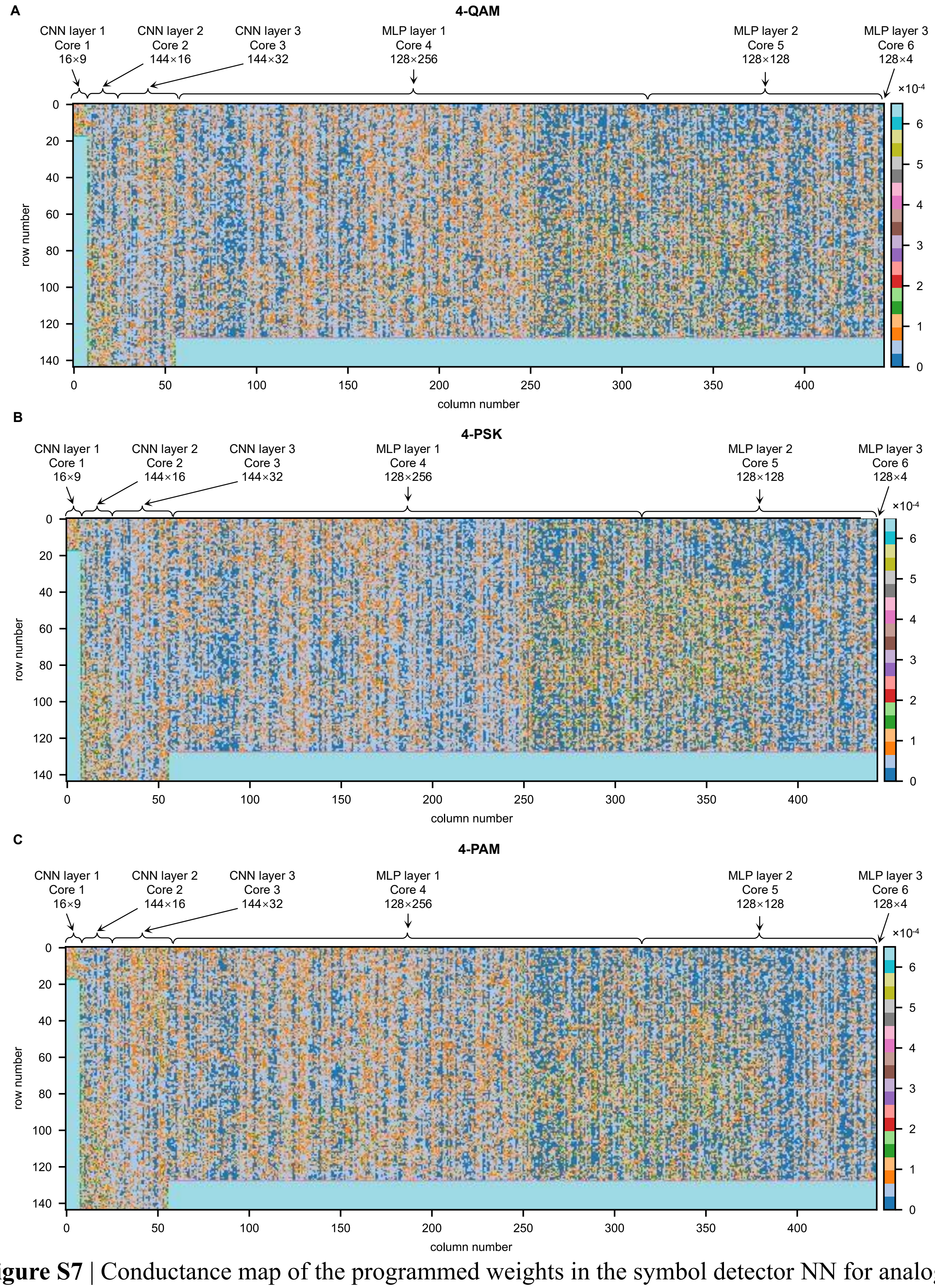

Figure S7 | Conductance map of the programmed weights in the symbol detector NN for analog signals modulated using different schemes.
A. 4-QAM. **B.** 4-PSK. **C.** 4-PAM.

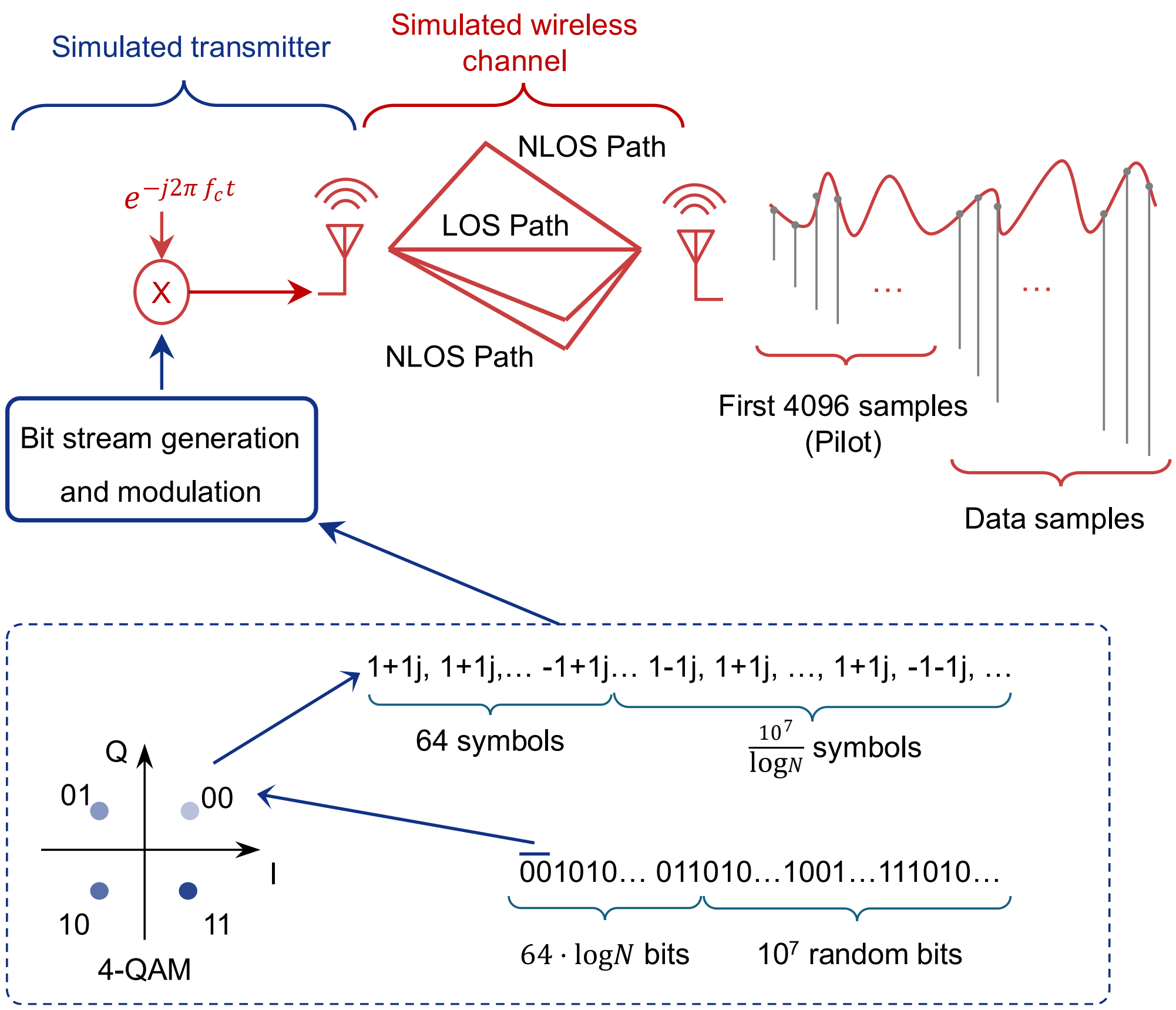

Figure S8 | The workflow of the simulated transmitter and wireless channel.
The first portion of the bitstream, consisting of $64 \cdot \log_2 N$ bits, is used as pilot symbols by repeating a specific "01" pattern. The number of pilot bits depends on the modulation scheme N, ensuring exactly 64 pilot symbols regardless of the modulation order. The symbols are then modulated onto a high-frequency carrier, and the real and imaginary components are combined before transmission through an antenna. The signal passes through a Rayleigh fading channel and is received as a distorted analog signal. The first 4096 received samples (64 samples per symbol) are aligned with the known pilot symbols for channel feature extraction. The remaining samples are used for direct symbol detection and recovery of the original information bits from the analog waveform.

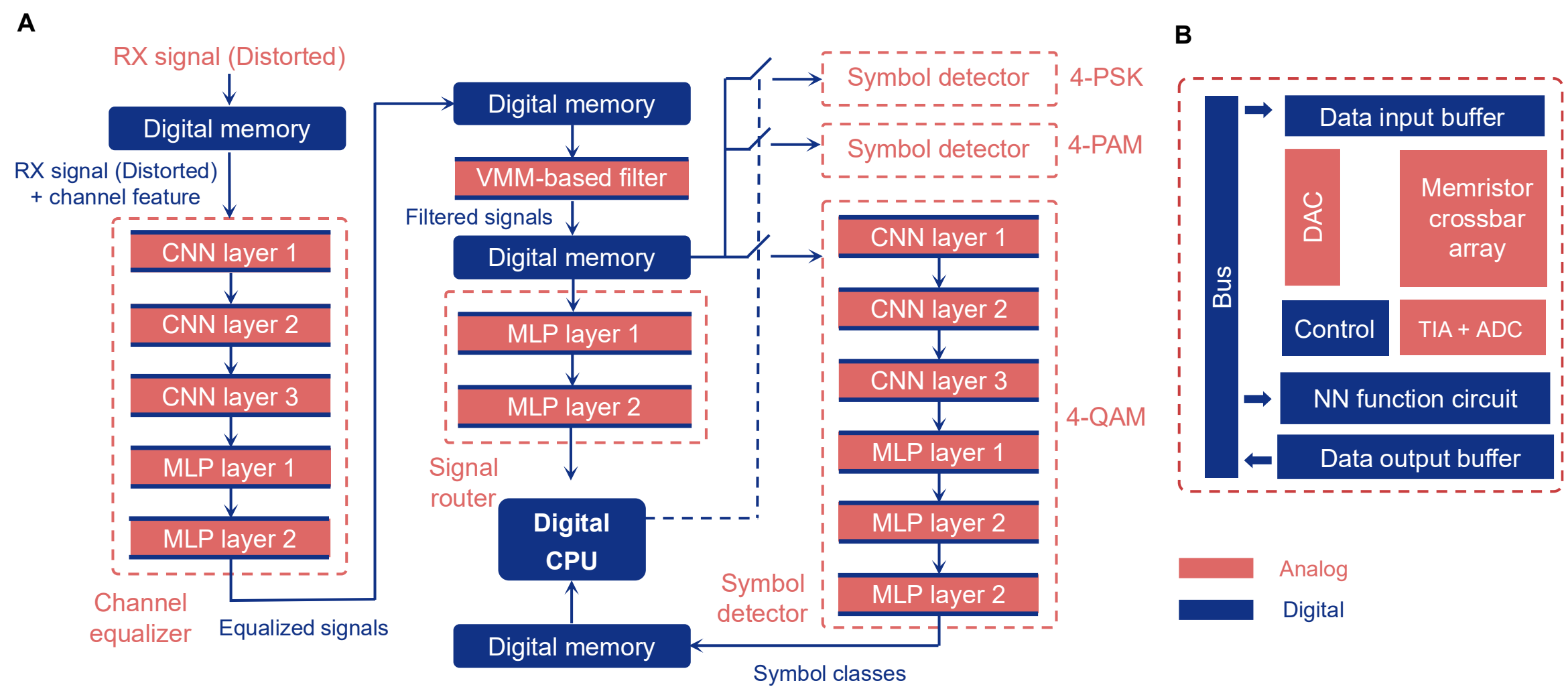

Figure S9 | Hardware workflow of the NN-based wireless receiver.

A. The detailed hardware workflow of the NN-based wireless receiver. **B.** The architecture of one memristive computing core with one memristor crossbar array, DAC, TIA, ADC, digital buffer, activation function, and control logic.

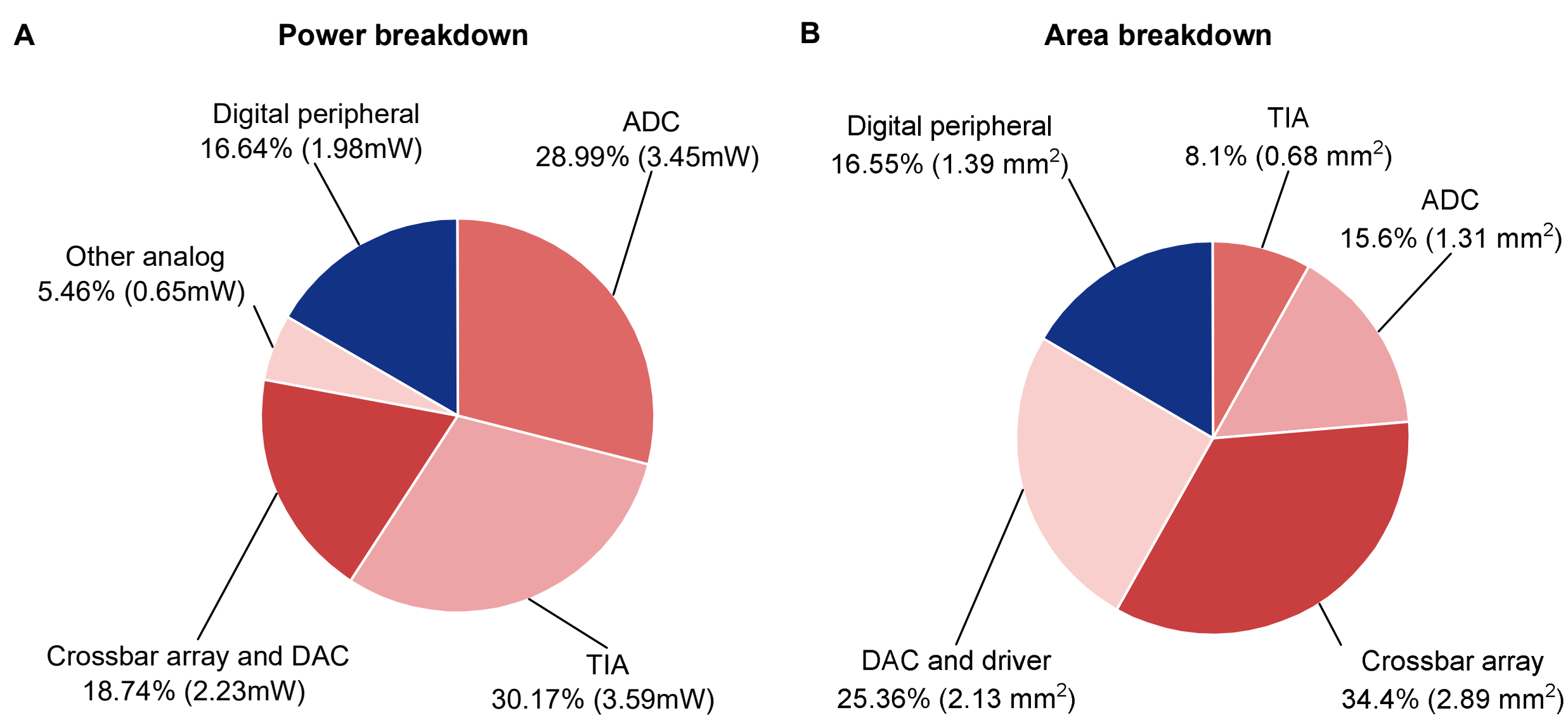

Figure S10 | Power and area breakdown of the memristive SoC.

A. The power breakdown of the memristive SoC. **B.** The area breakdown of the memristive SoC.

Supplementary Tables

Table S1 | Comparison with traditional signal processing methods

**Feature**	**NN-based signal processing**	**Traditional signal processing** (*23*, *24, 36, 37*)
Adaptability	High	Low
Robustness	High	Moderate
Channel estimation dependency	Not Required	Required
Scalability	High scalability	Limited scalability
Training required	Yes	No
Complexity	High	Moderate

Table S2 | Comparison with NN-based receivers on digital hardware

**Works**	**Hardware**	**Input signals**	**NN Architecture**	**Retraining steps to new environment**	**Energy efficiency**
Ref. (*55*)	GPU	Baseband	Monolithic deep NN	~3×10^4 steps	3.12 TOPS/W
Ref. (*9*)	GPU	Baseband	Monolithic deep NN	~ 10^3 – 10^5 steps	3.12 TOPS/W
Ref. (*56*)	Xilinx FPGA	Baseband	Monolithic deep NN	~10^3 steps	0.011 TOPS/W
This work	Memristive SoC	High-frequency RF	Modular task-specific NNs	~ 210 steps	21.3 TOPS/W

Table S3 | Comparison with DSP hardware

**Hardware platform**	**Device**	**Communication throughput**	**Energy efficiency**
DSP(*38*)	CMOS IC	3.968 Gbps	2.6 TOPS/W
DSP(*39*)	CMOS IC	0.0621 Gbps	0.02 TOPS/W
TPU(*41*)	CMOS IC	65.4 Gbps	0.8 TOPS/W
FPGA(*40*)	CMOS IC	1.16 Gbps	1.35 TOPS/W
This Work	Memristive SoC	3.968 Gbps	21.3 TOPS/W

Gbps: Gigabits per second. TOPS/W: tera operations per second per Watt.

Table S4 | Comparison with AIMC hardware for wireless signal processing

**Works**	**Ref. (*23*)**	**Ref. (*42*)**	**Ref. (*24*)**	**Ref. (*21*)**	**Ref. (*25*)**	**This Work**
**Technology node**	180 nm	N/A	180 nm	65 nm	40 nm	65 nm
**Signal processing**	Demodulation	Demodulation	Detection	Detection	Detection	Decoding
**Algorithm**	N/A	ADMM	MMSE	ZF	ZF	Neural networks
**Crossbar array size**	34×32	N/A	64×64	248×256	8×8	248×256
**Modulation**	4-QAM	64-QAM	16-QAM	16-PSK	256-QAM	4-QAM, 4-PAM, 4-PSK
**Experiment**	Experiment+ simulation	Simulation	Experiment + simulation	Full experiment	Experiment + simulation	Full experiment
**Energy efficiency**	222 TOPS/W	5.18 TOPS/W	N/A	21.3 TOPS/W	≤ 6 TOPS/W	21.3 TOPS/W

RZF: Regularized zero forcing. ZF: Zero forcing. ADMM: Alternating direction method of multipliers.

Table S5 | Adaptability of the NN-based wireless receiver

**Channel**	**Model**	**Equalizer MSE**	**BER**	**Retraining steps**
Rayleigh (baseline)	6-tap UMa-like, 30 Hz Doppler	2.45×10^{-4}	$< 3 \times 10^{-5}$	/
Rayleigh (mild drift)	Increased Doppler (40 Hz) and mild perturbations to path delays (±5%) and path gains (±0.5 dB)	2.2×10^{-5}	$< 3 \times 10^{-5}$	100
Rayleigh (drastic shift)	High Doppler (80 Hz) and completely reconfigured multipath delays and gains	2.3×10^{-5}	$< 3 \times 10^{-5}$	800
LoS	Rician fading channel (K = 10 dB) with a dominant	2.95×10^{-5}	$< 3 \times 10^{-5}$	220

	direct path and 5 Hz Doppler			
NLoS	CDL-A channel with 11 propagation paths and 50 Hz Doppler	1.86×10^{-7}	$< 3 \times 10^{-5}$	1340

Table S6 | Scalability of the NN-based wireless receiver

Scheme	SNR (dB)	Network weight size	BER (QAT)
16-QAM	20	~9.0×10^{4} (baseline)	$< 1\times10^{-4}$
64-QAM	20	~9.3×10^{4} (baseline)	2.7×10^{-3}
128-QAM	24	~5.5×10^{5} (enlarged, ~6×)	5.0×10^{-4}
256-QAM	28	~5.6×10^{5} (enlarged, ~6×)	8.0×10^{-4}

QAT: Quantization aware training

Table S7 | Signal Router NN Configuration

Layer	Shape	Params
FC1: Linear(64→128) + ReLU	64 → 128	8,320
FC2: Linear(128→128) + ReLU	128 → 128	16,512
FC3: Linear(128→7)	128 → 7	903
Total		25,735 (101 KB)

Table S8 | Channel Equalizer NN Configuration

Sub-module	Architecture	Params	Size
Pilot encoder	5× Conv2d + 2× FC	21,020	82 KB
Equalizer branch	3× Conv2d + 2× FC	84,064	328 KB
Full model		105,084	410 KB

Table S9 | Symbol Detector NN Configuration

Layer	Shape	Params
Conv1: Conv2d(1→8, 3×3) + ReLU + AvgPool2d(2)	(1, 8, 16) → (8, 4, 8)	80
Conv2: Conv2d(8→16, 3×3) + ReLU + AvgPool2d(2)	(8, 4, 8) → (16, 2, 4)	1,168
Conv3: Conv2d(16→16, 3×3)	(16, 2, 4) → (16, 2, 4)	2,320
Flatten	(16, 2, 4) → 128	—
FC1: Linear(128→128) + ReLU	128 → 128	16,512
FC2: Linear(128→64) + ReLU	128 → 64	8,256
FC3: Linear(64→N)	64 → N (symbols/scheme)	260*
Total		28,596 (112 KB)

Table S10 | NN Configuration Summary

Module	Params	Role
Signal router (MLP)	25,735	Modulation classification (7 classes)
Equalizer (full)	105,084	Software training
Equalizer_HW (deployed)	84,064	Crossbar deployment
Equalizer FC retrain	82,560	Hardware noise adaptation (FC layers only)
Symbol detector	28,596	Symbol classification
Deployed system total	138,395	Router + Equalizer_HW + Detector

REFERENCES

1. K. Burse, R. N. Yadav, S. C. Shrivastava, Channel equalization using neural networks: A review. *IEEE Trans. Syst. Man Cybern. C Appl. Rev.* **40**, 352–357 (2010).

2. D. F. Carrera, C. Vargas-Rosales, N. M. Yungaicela-Naula, L. Azpilicueta, Comparative study of artificial neural network based channel equalization methods for mmWave communications. *IEEE Access* **9**, 41678–41687 (2021).

3. J. Rock, W. Roth, M. Toth, P. Meissner, F. Pernkopf, Resource-efficient deep neural networks for automotive radar interference mitigation. *IEEE J. Sel. Top. Signal Process.***15**, 927–940 (2021).

4. H. Sun, X. Chen, Q. Shi, M. Hong, X. Fu, N. D. Sidiropoulos, "Learning to optimize: Training deep neural networks for wireless resource management," in *2017 IEEE 18th International Workshop on Signal Processing Advances in Wireless Communications (SPAWC)* (IEEE, 2017), pp. 1–6.

5. X. Liu, D. Yang, A. El Gamal, "Deep neural network architectures for modulation classification," in *2017 51st Asilomar Conference on Signals, Systems, and Computers* (IEEE, 2017), pp. 915–919.

6. K. Tsagkaris, A. Katidiotis, P. Demestichas, Neural network-based learning schemes for cognitive radio systems. *Comput. Commun.* **31**, 3394–3404 (2008).

7. M. Khasawneh, A. Azab, S. Alrabaee, H. Sakkal, H. H. Bakhit, Convergence of IoT and cognitive radio networks: A survey of applications, techniques, and challenges. *IEEE Access* **11**, 71097–71112 (2023).

8. K. M. Cohen, S. Park, O. Simeone, S. Shamai Shitz, Calibrating AI models for wireless communications via conformal prediction. *IEEE Trans. Mach. Learn. Commun. Netw.* **1**, 296–312 (2023).

9. R. Wiesmayr, S. Cammerer, F. A. Aoudia, J. Hoydis, J. Zakrzewski, A. Keller, "Design of a standard-compliant real-time neural receiver for 5G NR," in *2025 IEEE International*

Conference on Machine Learning for Communication and Networking (ICMLCN) (IEEE, 2025), pp. 1–6.

10. F. Ait Aoudia, J. Hoydis, End-to-end learning for ofdm: From neural receivers to pilotless communication. *IEEE Trans. Wirel. Commun.* **21**, 1049–1063 (2022).

11. S. Cammerer, F. A. Aoudia, J. Hoydis, A. Oeldemann, A. Roessler, T. Mayer, A. Keller, "A neural receiver for 5G NR multi-user MIMO," in *2023 IEEE Globecom Workshops (GC Wkshps)* (IEEE, 2023), pp. 329–334.

12. H. He, H. Jiang, Deep learning based energy efficiency optimization for distributed cooperative spectrum sensing. *IEEE Wirel. Commun.* **26**, 32–39 (2019).

13. Y. Huang, T. Ando, A. Sebastian, M.-F. Chang, J. J. Yang, Q. Xia, Memristor-based hardware accelerators for artificial intelligence. *Nat. Rev. Electr. Eng.* **1**, 286–299 (2024).

14. C. Li, D. Belkin, Y. Li, P. Yan, M. Hu, N. Ge, H. Jiang, E. Montgomery, P. Lin, Z. Wang, J. P. Strachan, M. Barnell, Q. Wu, R. S. Williams, J. J. Yang, Q. Xia, "In-memory computing with memristor arrays," in *2018 IEEE International Memory Workshop (IMW)* (IEEE, 2018), pp. 1–4.

15. M. Prezioso, F. Merrikh-Bayat, B. D. Hoskins, G. C. Adam, K. K. Likharev, D. B. Strukov, Training and operation of an integrated neuromorphic network based on metal-oxide memristors. *Nature* **521**, 61–4 (2015).

16. Q. Xia, J. J. Yang, Memristive crossbar arrays for brain-inspired computing. *Nat. Mater.* **18**, 309–323 (2019).

17. F. Kiani, J. Yin, Z. Wang, J. J. Yang, Q. Xia, A fully hardware-based memristive multilayer neural network. *Sci. Adv.* **7**, 4801 (2021).

18. S. Ambrogio, P. Narayanan, A. Okazaki, A. Fasoli, C. Mackin, K. Hosokawa, A. Nomura, T. Yasuda, A. Chen, A. Friz, M. Ishii, J. Luquin, Y. Kohda, N. Saulnier, K. Brew, S. Choi, I. Ok, T. Philip, V. Chan, C. Silvestre, I. Ahsan, V. Narayanan, H. Tsai, G. W. Burr, An analog-AI chip for energy-efficient speech recognition and transcription. *Nature* **620**, 768–775 (2023).

19. H. Jiang, L. Han, P. Lin, Z. Wang, M. H. Jang, Q. Wu, M. Barnell, J. J. Yang, H. L. Xin, Q. Xia, Sub-10 nm Ta channel responsible for superior performance of a HfO_2 memristor. *Sci. Rep.* **6**, 28525 (2016).

20. C. Li, M. Hu, Y. Li, H. Jiang, N. Ge, E. Montgomery, J. Zhang, W. Song, N. Dávila, C. E. Graves, Z. Li, J. P. Strachan, P. Lin, Z. Wang, M. Barnell, Q. Wu, R. S. Williams, J. J. Yang, Q. Xia, Analogue signal and image processing with large memristor crossbars. *Nat. Electron.* **1**, 52–59 (2018).

21. Y. Huang, C. He, Y. Ling, N. Ge, J. Joshua Yang, M. Hu, L. Katehi, Q. Xia, Radiofrequency signal processing with a memristive system-on-a-chip. *Nat. Electron.* **8**, 587–596 (2025).

22. Z. Wang, W. Song, T. Wang, Z. Wang, Y. Xu, M. Rao, F. Cai, W. Yin, M. S.-W. Chen, N. Ge, M. Collins, K. Bai, S. Ganguli, M. R. Page, Q. Wu, L. Katehi, Q. Xia, M. Hu, J. J. Yang, Real-time signal processing enabled by fused networks on a memristor-based system on a chip. *Sci. Adv.* **11**, 3436 (2025).

23. C. Wang, G.-J. Ruan, Z.-Z. Yang, X.-J. Yangdong, Y. Li, L. Wu, Y. Ge, Y. Zhao, C. Pan, W. Wei, L.-B. Wang, B. Cheng, Z. Zhang, C. Zhang, S.-J. Liang, F. Miao, Parallel in-memory wireless computing. *Nat. Electron.* **6**, 381–389 (2023).

24. Q. Zeng, J. Liu, M. Jiang, J. Lan, Y. Gong, Z. Wang, Y. Li, C. Li, J. Ignowski, K. Huang, Realizing in-memory baseband processing for ultrafast and energy-efficient 6G. *IEEE Internet Things J.* **11**, 5169–5183 (2024).

25. P. Zuo, Q. Wang, Y. Luo, R. Xie, S. Wang, Z. Cheng, L. Bao, Z. Wang, Y. Cai, R. Huang, Z. Sun, Precise and scalable analogue matrix equation solving using resistive random-access memory chips. *Nat. Electron.* **8**, 1222–1233 (2025).

26. S. Haykin, Cognitive radio: Brain-empowered wireless communications. *IEEE J. Sel. Areas Commun.* **23**, 201–220 (2005).

27. Q. Zhang, H. Dong, J. Zhao, Channel estimation for high-speed railway wireless communications: A generative adversarial network approach. *Electronics* **12**, 1752 (2023).

28. D. Gomez, M. Sroka, J. L. Gonzalez Gonzalez Jimenez, Process and temperature compensation for RF low-noise amplifiers and mixers. *IEEE Trans. Circuits Syst. I Reg. Pap.* **57**, 1204–1211 (2010).

29. S. Lee, J. Kim, K. Lee, H.-J. Song, 248-GHz subharmonic mixer last transmitter with I/Q imbalance and LO feedthrough calibration. *IEEE Solid. State. Circuits Lett.* **7**, 159–162 (2024).

30. J. M. Cioffi, G. P. Dudevoir, M. Vedat Eyuboglu, G. D. Forney, MMSE decision-feedback equalizers and coding. I. Equalization results. *IEEE Trans. Commun.* **43**, 2582–2594 (1995).

31. N. Farsad, A. Goldsmith, Neural network detection of data sequences in communication systems. *IEEE Trans. Signal Process.* **66**, 5663–5678 (2018).

32. Z. Zhou, L. Liu, H.-H. Chang, Learning for detection: MIMO-OFDM symbol detection through downlink pilots. *IEEE Trans. Wirel. Commun.* **19**, 3712–3726 (2020).

33. Y. Liao, N. Farsad, N. Shlezinger, Y. C. Eldar, A. J. Goldsmith, "Deep neural network symbol detection for millimeter wave communications," in *2019 IEEE Global Communications Conference (GLOBECOM)* (IEEE, 2019), pp. 1–6.

34. K. Hacioglu, An improved recurrent neural network for M-PAM symbol detection. *IEEE Trans. Neural Netw.* **8**, 779–783 (1997).

35. M. Abdul Aziz, M. H. Rahman, M. Abrar Shakil Sejan, R. Tabassum, D.-D. Hwang, H.-K. Song, Deep recurrent neural network based detector for OFDM with index modulation. *IEEE Access* **12**, 89538–89547 (2024).

36. C.-L. Xiong, D.-G. Wang, X.-Y. Zhang, J.-B. Wei, C.-J. Tang, "Recursive channel estimation algorithms for iterative receiver in MIMO-OFDM systems," in *2009 IEEE Wireless Communications and Networking Conference* (IEEE, 2009), pp. 1–5.

37. L. Sanguinetti, E. Bjornson, J. Hoydis, Toward massive MIMO 2.0: Understanding spatial correlation, interference suppression, and pilot contamination. *IEEE Trans. Commun.* **68**, 232–257 (2020).

38. A. Agarwal, S. K. Mathew, S. K. Hsu, M. A. Anders, H. Kaul, F. Sheikh, R. Ramanarayanan, S. Srinivasan, R. Krishnamurthy, S. Borkar, "A 320mV-to-1.2V on-die fine-grained reconfigurable fabric for DSP/media accelerators in 32nm CMOS," in *2010 IEEE International Solid-State Circuits Conference - (ISSCC)* (IEEE, 2010), pp. 328–329.

39. T. Fryza, R. Mego, "Power consumption of multicore digital signal processor: Theoretical analysis and real applications," in *2014 IEEE 23rd International Symposium on Industrial Electronics (ISIE)* (IEEE, 2014), pp. 1894–1898.

40. E. Taka, D. Gourounas, A. Gerstlauer, D. Marculescu, A. Arora, "Efficient approaches for GEMM acceleration on leading AI-optimized FPGAs", in *2024 IEEE 32nd Annual International Symposium on Field-Programmable Custom Computing Machines (FCCM)* (IEEE, 2024), pp. 54–65.

41. N. Jouppi, G. Kurian, S. Li, P. Ma, R. Nagarajan, L. Nai, N. Patil, S. Subramanian, A. Swing, B. Towles, C. Young, X. Zhou, Z. Zhou, D. A. Patterson, "TPU v4: An Optically Reconfigurable Supercomputer for Machine Learning with Hardware Support for Embeddings," in *Proceedings of the 50th Annual International Symposium on Computer Architecture* (ACM, New York, NY, USA, 2023), pp. 1–14.

42. J.-H. Bi, S. Yang, P. Zhang, High-speed and ultra-energy-efficient in-memory computing circuit for ADMM-Based box-constrained massive MIMO signal detection. *IEEE Wirel. Commun. Lett.* **14**, 3530–3534 (2025).

43. M. Le Gallo, R. Khaddam-Aljameh, M. Stanisavljevic, A. Vasilopoulos, B. Kersting, M. Dazzi, G. Karunaratne, M. Brändli, A. Singh, S. M. Müller, J. Büchel, X. Timoneda, V. Joshi, M. J. Rasch, U. Egger, A. Garofalo, A. Petropoulos, T. Antonakopoulos, K. Brew, S. Choi, I. Ok, T. Philip, V. Chan, C. Silvestre, I. Ahsan, N. Saulnier, V. Narayanan, P. A. Francese, E. Eleftheriou, A. Sebastian, A 64-core mixed-signal in-memory compute chip based on phase-change memory for deep neural network inference. *Nat. Electron.* **6**, 680–693 (2023).

44. W. Song, M. Rao, Y. Li, C. Li, Y. Zhuo, F. Cai, M. Wu, W. Yin, Z. Li, Q. Wei, S. Lee, H. Zhu, L. Gong, M. Barnell, Q. Wu, P. A. Beerel, M. S.-W. Chen, N. Ge, M. Hu, Q. Xia, J. J. Yang,

Programming memristor arrays with arbitrarily high precision for analog computing. *Science* **383**, 903–910 (2024).

45. L. T. Chabane, D.-K. G. Pham, P. Chollet, P. Desgreys, “Design method of analog sigmoid function and its approximate derivative” in *2021 XXXVI Conference on Design of Circuits and Integrated Systems (DCIS)* (IEEE, 2021), pp. 1–5.

46. C. Geng, Q. Sun, S. Nakatake, “An analog CMOS implementation for multi-layer perceptron with ReLU activation” in *2020 9th International Conference on Modern Circuits and Systems Technologies (MOCAST)* (IEEE, 2020), pp. 1–6.

47. W. Zhang, P. Yao, B. Gao, Q. Liu, D. Wu, Q. Zhang, Y. Li, Q. Qin, J. Li, Z. Zhu, Y. Cai, D. Wu, J. Tang, H. Qian, Y. Wang, H. Wu, Edge learning using a fully integrated neuro-inspired memristor chip. *Science* **381**, 1205–1211 (2023).

48. C. Li, Z. Wang, M. Rao, D. Belkin, W. Song, H. Jiang, P. Yan, Y. Li, P. Lin, M. Hu, N. Ge, J. P. Strachan, M. Barnell, Q. Wu, R. S. Williams, J. J. Yang, Q. Xia, Long short-term memory networks in memristor crossbar arrays. *Nat. Mach. Intell.* **1**, 49–57 (2019).

49. M. Rao, H. Tang, J. Wu, W. Song, M. Zhang, W. Yin, Y. Zhuo, F. Kiani, B. Chen, X. Jiang, H. Liu, H.-Y. Chen, R. Midya, F. Ye, H. Jiang, Z. Wang, M. Wu, M. Hu, H. Wang, Q. Xia, N. Ge, J. Li, J. J. Yang, Thousands of conductance levels in memristors integrated on CMOS. *Nature* **615**, 823–829 (2023).

50. C. Li, D. Belkin, Y. Li, P. Yan, M. Hu, N. Ge, H. Jiang, E. Montgomery, P. Lin, Z. Wang, W. Song, J. P. Strachan, M. Barnell, Q. Wu, R. S. Williams, J. J. Yang, Q. Xia, Efficient and self-adaptive in-situ learning in multilayer memristor neural networks. *Nat. Commun.* **9**, 2385 (2018).

51. Z. Wang, C. Li, P. Lin, M. Rao, Y. Nie, W. Song, Q. Qiu, Y. Li, P. Yan, J. P. Strachan, N. Ge, N. Mcdonald, Q. Wu, M. Hu, H. Wu, R. S. Williams, Q. Xia, J. J. Yang, In situ training of feed-forward and recurrent convolutional memristor networks. *Nat. Mach. Intell.* **1**, 434–442 (2019).

52. K. He, X. Zhang, S. Ren, J. Sun, “Deep residual learning for image recognition” in *IEEE Conference on Computer Vision and Pattern Recognition (CVPR)* (2016); http://image-net.org/challenges/LSVRC/2015/.

53. A. Pinkus, Approximation theory of the MLP model in neural networks. *Acta Numerica* **8**, 143–195 (1999).

54. T. O. Hodson, Root-mean-square error (RMSE) or mean absolute error (MAE): When to use them or not. *Geosci. Model Dev.* **15**, 5481–5487 (2022).

55. M. Honkala, D. Korpi, J. M. J. Huttunen, DeepRx: Fully convolutional deep learning receiver. *IEEE Trans. Wirel. Commun.* **20**, 3925–3940 (2021).

56. J. Ney, N. Wehn, “Achieving high throughput with a trainable neural-network-based equalizer for communications on FPGA” in *2024 27th Euromicro Conference on Digital System Design (DSD)* (IEEE, 2024), pp. 106–113.

57. “IEEE Standard for Information Technology- Telecommunications and Information Exchange Between Systems- Local and Metropolitan Area Networks- Specific Requirements Part Ii: Wireless LAN Medium Access Control (MAC) and Physical Layer (PHY) Specifications” (IEEE, Piscataway, NJ, USA, 2003); https://doi.org/10.1109/IEEESTD.2003.94282.
